# A Workflow for Identifying Viable Crystal Structures with Partially Occupied Sites Applied to the Solid Electrolyte Cubic Li_7_La_3_Zr_2_O_12_

**DOI:** 10.1021/acs.jpclett.3c02064

**Published:** 2023-11-08

**Authors:** Julian Holland, Tom Demeyere, Arihant Bhandari, Felix Hanke, Victor Milman, Chris-Kriton Skylaris

**Affiliations:** † School of Chemistry, University of Southampton, Southampton SO17 1BJ, U.K.; ‡ The Faraday Institution, Quad One, Becquerel Avenue, Harwell Campus, Didcot OX11, U.K.; ¶ BIOVIA, 22 Cambridge Science Park, Milton Road, Cambridge CB4 0FJ, U.K.

## Abstract

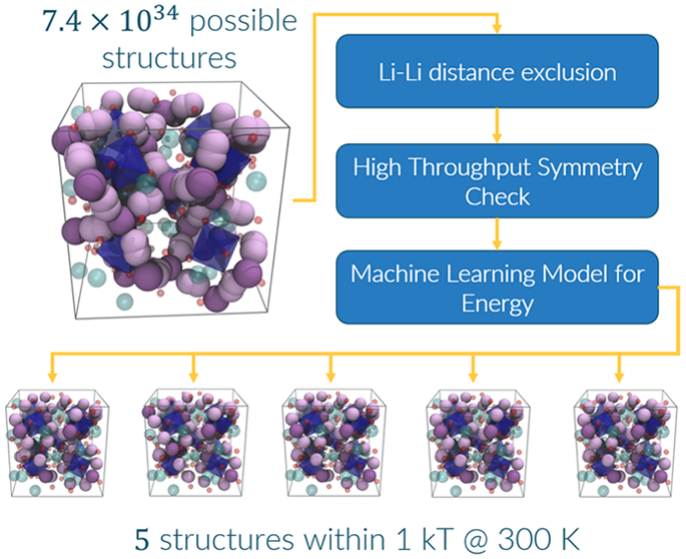

To date, experimental and theoretical works have been unable to uncover the ground-state configuration of the solid electrolyte cubic Li_7_La_3_Zr_2_O_12_ (*c*-LLZO). Computational studies rely on an initial low-energy structure as a reference point. Here, we present a methodology for identifying energetically favorable configurations of *c*-LLZO for a crystallographically predicted structure. We begin by eliminating structures that involve overlapping Li atoms based on nearest neighbor counts. We further reduce the configuration space by eliminating symmetry images from all remaining structures. Then, we perform a machine learning-based energetic ordering of all remaining structures. By considering the geometrical constraints that emerge from this methodology, we determine that a large portion of previously reported structures may not be feasible or stable. The method developed here could be extended to other ion conductors. We provide a database containing all of the generated structures with the aim of improving accuracy and reproducibility in future *c*-LLZO research.

The use of solid electrolytes in Li-ion batteries promises higher energy densities, improved safety, longer lifetimes,^1^ and reduced production costs compared to the current generation of commercial liquid electrolyte cells.^2,3^ However, poor electrochemical and chemomechanical stability present a significant issue for a number of superionic solid-state conductors;^4^ for example, dendrite formation through the electrolyte, which causes batteries to short-circuit, has been reported even for the most stable solid electrolytes.^5−9^ Li_7_La_3_Zr_2_O_12_ (LLZO) has the best interfacial stability out of all of the popular fast ion conducting solid electrolytes against metallic Li.^10^ LLZO has two primary polymorphs: the highly conducting disordered cubic LLZO (*c*-LLZO) and the ordered tetragonal LLZO (*t*-LLZO), which has a Li diffusivity that is more than 2 orders of magnitude smaller than that of the cubic phase.^11,12^*c*-LLZO has a Hermann–Mauguin space group of *Ia*3̅*d* with Li atoms partially occupying the 24*d* and 96*h* sites (Figure 1b). *c*-LLZO is not stable at room temperature, but it has been stabilized with a number of substitutional dopants on its 24*d* sites.^13−16^ The 24*d* site is tetrahedral, and the 96*h* site occurs at a tetrahedral/octahedral interface.

**Figure 1:**
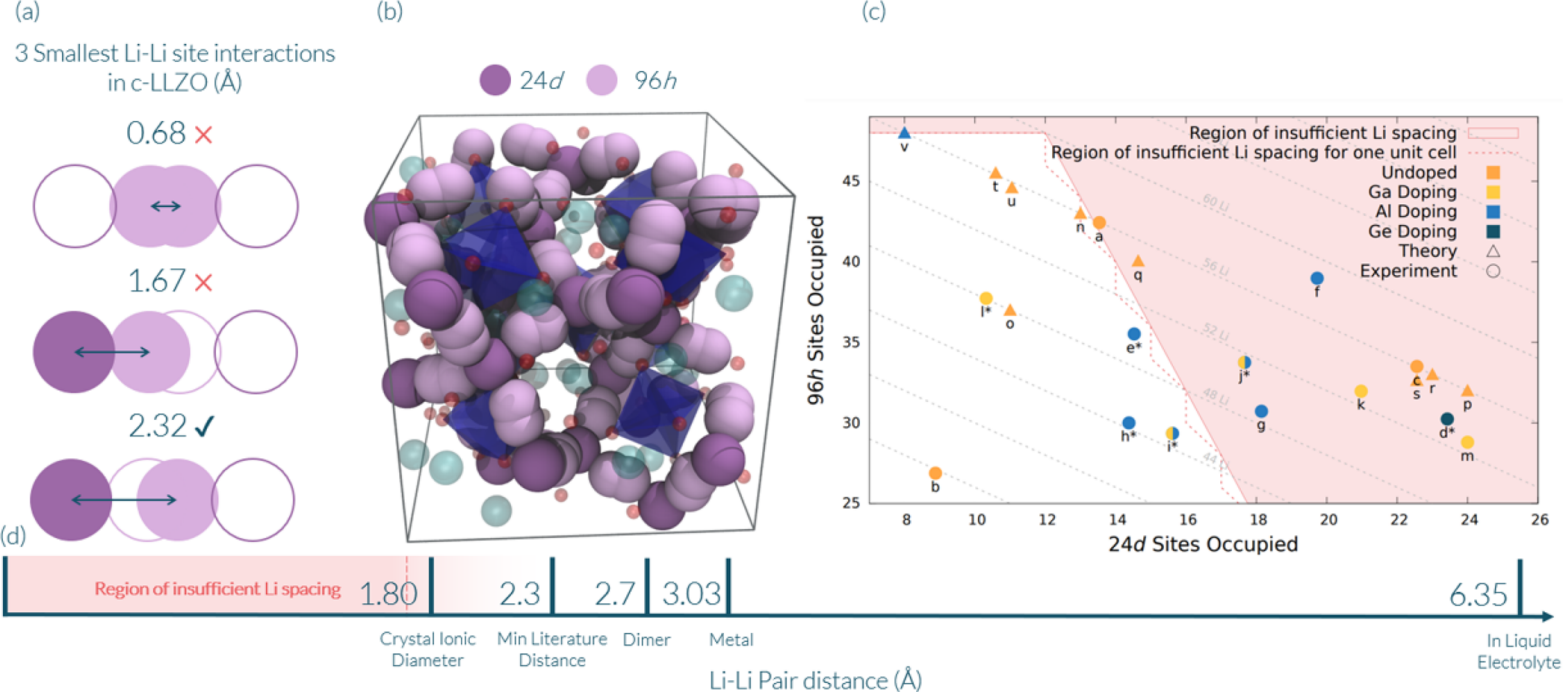
(a) Examples of the three shortest site interactions from the center of each atom. The first and second neighboring Li sites are too close and cannot simultaneously contain a Li atom. (b) Unit cell of *c*-LLZO with all of the 24*d* (dark purple) and 96*h* (light purple) sites highlighted. O and La atoms are represented by red and green spheres, respectively, and Zr atoms are represented by blue polyhedra. (c) Experimental and theoretical site assignments and/or starting structures in the literature: a,^17^ b,^13^ c,^12^ d,^18^ e,^19^ f,^20^ g,^21^ h,^22^ i,^22^ j,^22^ k,^23^ l,^24^ m,^21^ n,^7−9,25−29^ o,^30^ p,^31,32^ q,^33^ r,^34^ s,^35^ t,^35^ and u.^36^ Asterisks (*) indicate that the experimental assignments used neutron diffraction; the rest of the structures were solved with X-ray diffraction. The region in red indicates the 24*d*:96*h* ratios that cannot exist without a Li–Li interaction below the crystal ionic diameter. The data that were used for this image are provided in the Supporting Information (Tables S1 and S2). (d) A scale showing important Li–Li interaction distances that have been recorded in the literature: the crystal ionic diameter,^37^ min literature distance,^38^ metal,^39^ dimer (in a vacuum) (cf. Figure S6), and average Li–Li distance in a liquid electrolyte,^40^ as well as our chosen cutoff point at 1.7 Å (red dashed line).

Despite the technological relevance and intensive study^36,41−45^ of *c*-LLZO, there is no consensus of its Li occupancy (Figure 1c). Here, we present a combinatorial study to find and rank all possible Li site occupancies. Previous computational studies have often used one of the experimental values in Figure 1 as a starting point to obtain an idealized unit cell using a variety of methods (see Table S2). The range of predicted structures in the literature indicates some disagreement about the ground state. Without knowing whether the structures that are used are close to the ground state, more complicated thermodynamic and observable properties that are calculated from these structures may not be well-described.

To find the most stable structures, we intend to produce all possible structures of a *c*-LLZO unit cell and order them energetically to ascertain the presence of a significant energy difference in the choice of the structure. We also hope to elicit whether some of the structural properties, such as the 24*d*:96*h* ratio, are indicative of a structure’s energy. We do not include the 48*g* site that occurs between the 96*h* sites, as it is not reported in most of the refinements we found (see Table S1 for references). The structures produced in this work are to be published alongside our results (see Section S1 in the Supporting Information (SI)) in the hope that we can provide further clarity and improve reproducibility in this field of research.

The number of ways to populate 120 sites with 56 Li is , rendering a brute-force combinatorial method unviable. To reduce this configuration space, we begin by eliminating all overlapping Li atoms (see Figure 1a)^46^ by imposing a minimal Li–Li distance. Here, we use a distance of 1.7 Å (see Section S9), which is less than 2 times the ionic radius of a Li atom, smaller than the minimum distance in *t*-LLZO (2.56 Å),^47^ and significantly less than the Li–Li distances in metallic Li. In practice, any minimal distance between 1.67 and 2.3 Å would be suitable for a numerical implementation.

This constraint on allowable Li–Li distances immediately limits the physically possible 24*d*:96*h* ratios. To describe the possible structures available under this simple geometric constraint, we outline the two major consequences this limit imposes.

1. A maximum of 48 of the 96*h* sites can be occupied at any one time: each 96*h* site exists as a pair within 0.68 Å of each other, so at most, one site can contain a Li atom.
2. Each 24*d* site occupation eliminates four of the 96*h* sites, as they are within 1.67 Å of this site.

We can use this information to narrow the range of Li site occupations with the following equations:
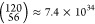
1
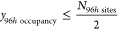
2where *N*_96*h* sites_ is the number of total 96*h* sites, *y*_96*h* occupancy_ is the number of occupied 96*h* sites, and *x*_24*d* occupancy_ is the number of occupied 24*d* sites. The area described in eqs 1 and 2 (i.e., the unshaded area in Figure 1c) is the region with sufficient Li spacing at a given Li concentration. The full region is shown in Figure S1. Outside of this area, the structures (by necessity) will have a Li–Li interaction that is smaller than what we would expect to find in reality. For the stoichiometrically predicted case (56 Li atoms per unit cell), we observe that 8 ≤ *x*_24*d* occupancy_ ≤ 13.3. This means that for a single unit cell, the only stoichiometric 24*d*:96*h* ratios that are possible are 8:48, 9:47, 10:46, 11:45, 12:44, and 13:43. This is how we refer to specific ratios throughout the rest of this Letter.

Figure 1c shows that a significant number of studies have reported structures that imply overlapping Li atoms with Li–Li distances smaller than 1.7 Å. For example, a structure with a 24*d*:96*h* ratio of 17:39 has a population of 17 occupied 24*d* sites, which necessitates that 68 (4 × 17) of the 96*h* sites fall within 1.7 Å of an occupied 24*d* site. Trying to distribute 39 Li atoms among the remaining 28 (96 – 68) sufficiently spaced 96*h* sites is impossible; thus, 11 of these Li atoms would occupy sites within 1.7 Å of another Li atom.

Having established the possible 24*d*:96*h* ratios, we could then generate all of the allowed structures. We initially generated all of the possible 24*d* permutations for a given 24*d* occupancy, eliminating directly neighboring 96*h* sites from consideration. We then populated the remaining 96*h* sites such that no two nearest neighbors were occupied simultaneously. The number of structures for each 24*d*:96*h* ratio (for one unit cell) is given in Table 1. We used the crystal structure sites reported by Buschmann et al.^19^ as the framework to perform our generation in.

**Table 1:** Total Number of *c*-LLZO Structures for Every Possible 24*d*:96*h* Ratio after Limiting to the Specific 24*d*:96*h* Ratio(s) (SI, Section S5), Mandating That All Li–Li Interactions Must Be >1.7 Å, and Ensuring the Generated Structures Are Symmetrically Unique

24*d*:96*h*	ratio limited	>1.7 Å spacing	symmetry unique
8:48	1.8 × 10^25^	80 019 456	816 454
9:47	9.3 × 10^23^	98 304 000	905 216
10:46	4.4 × 10^22^	41 754 624	366 971
11:45	1.9 × 10^21^	7 176 192	65 958
12:44	7.6 × 10^19^	427 176	4162
13:43	2.6 × 10^18^	1056	11
total	1.9 × 10^25^	227 682 504	2 158 772

This method generated all possible structures, approximately 2.3 × 10^8^, including duplicates having equivalent symmetries. To further reduce our large data set to unique conformations, we constructed the upper triangular connectivity matrix of the Li sublattice for each structure. First, we defined a connectivity matrix as the distances between each Li atom to every other Li atom. The matrix was then flattened and sorted to ensure uniformity in all of the matrices. This preprocessing of our structures decreased the memory required to store them, especially compared to that which is required for the full atomic structure of the crystal. A direct comparison of all the structures to check the symmetry would be computationally costly. Thus, we used local sensitive hashing (LSH)^48^ to perform fast approximate similarity searches with further lower memory requirements than the unhashed data. The use of LSH optimizes the pairwise comparison from scaling at  to , where *N* is the number of structures. This symmetry comparison method is designed to work for large data sets of similar data, which allowed us to perform symmetry checks on over 100 million structures in a reasonable time frame and with attainable memory requirements, something that would be impractical to attempt with conventional methods.^49−51^ The trade-off for increased speed and lower memory costs is the high specificity of the method. In practice, this method is limited to the comparison of monatomic sublattices with predefined positions.

We validated this method for both the 13:43 and 12:44 ratios (see the SI for further discussion). By employing this technique, we found that only ∼1% of our structures were symmetrically unique, allowing for a reduction by a further factor of ∼100 (see Table 1).

Having reduced the configuration space to around 2 × 10^6^ structures (Table 1), we were then able to energetically order our structures. To achieve this in a reasonable time frame, we performed density functional theory (DFT) calculations on small subsets of the total number of structures. The results were then used to fit a multiple linear regression (MLR) model. The DFT calculations were performed on two major subsets of the structures. The first subset was the 200 energetically lowest structures, according to the COMPASS III force field^52^ (see Section S6). The second subset was a random selection of approximately 1000 structures across the entire configuration space to ensure good coverage. In total, 1235 single-point DFT calculations were performed.

All of the DFT calculations were performed with the ONETEP code, where the computational cost scales linearly with the number of atoms, as opposed to the cubic scaling in conventional DFT.^53,54^ We used the PBE GGA exchange correlation functional^55^ and a kinetic energy cutoff of 830 eV. Further details, including all of the input files, can be found in the SI, Sections S1 and S7.

Because some structures had Li distributed in such a way that nonidentity symmetry operations were possible, there was variation in the contribution of configurational entropy to the total energy. We calculated the configurational entropy for all structures and have included it in all results presented in this work, unless stated otherwise. A discussion of how we calculated the configurational entropy is provided in Section S11 of the SI.

Because we are working with only crystallographically predicted structures, the base LaZrO structure remains unchanged with each configuration; therefore all energetic changes are due to the placement of Li. We acknowledge that in reality, the geometry does change considerably depending on the Li environment.^32^ However, this is a suitable assumption to make to energetically order the crystallographically predicted structures we generated. Therefore, an expression of all types of Li interactions would be a sufficient descriptor for predicting the energies of *c*-LLZO structures. The DFT energies were paired with a numerical representation of the structure and fit using a MLR model. The structures were represented by a frequency occurrence list of all possible Li–Li interactions. This representation is a reformulation of the connectivity matrix of the Li sublattice that was used for the symmetry reduction described above. The data were split into test and training sets with a 1:3 ratio. For further details regarding this methodology, we refer the reader to Section S8 of the SI.

By applying this model to our test set, we found a correlation of 0.9996 (see Figure 2a) with DFT energies with a mean average error of 0.0325 eV.

**Figure 2:**
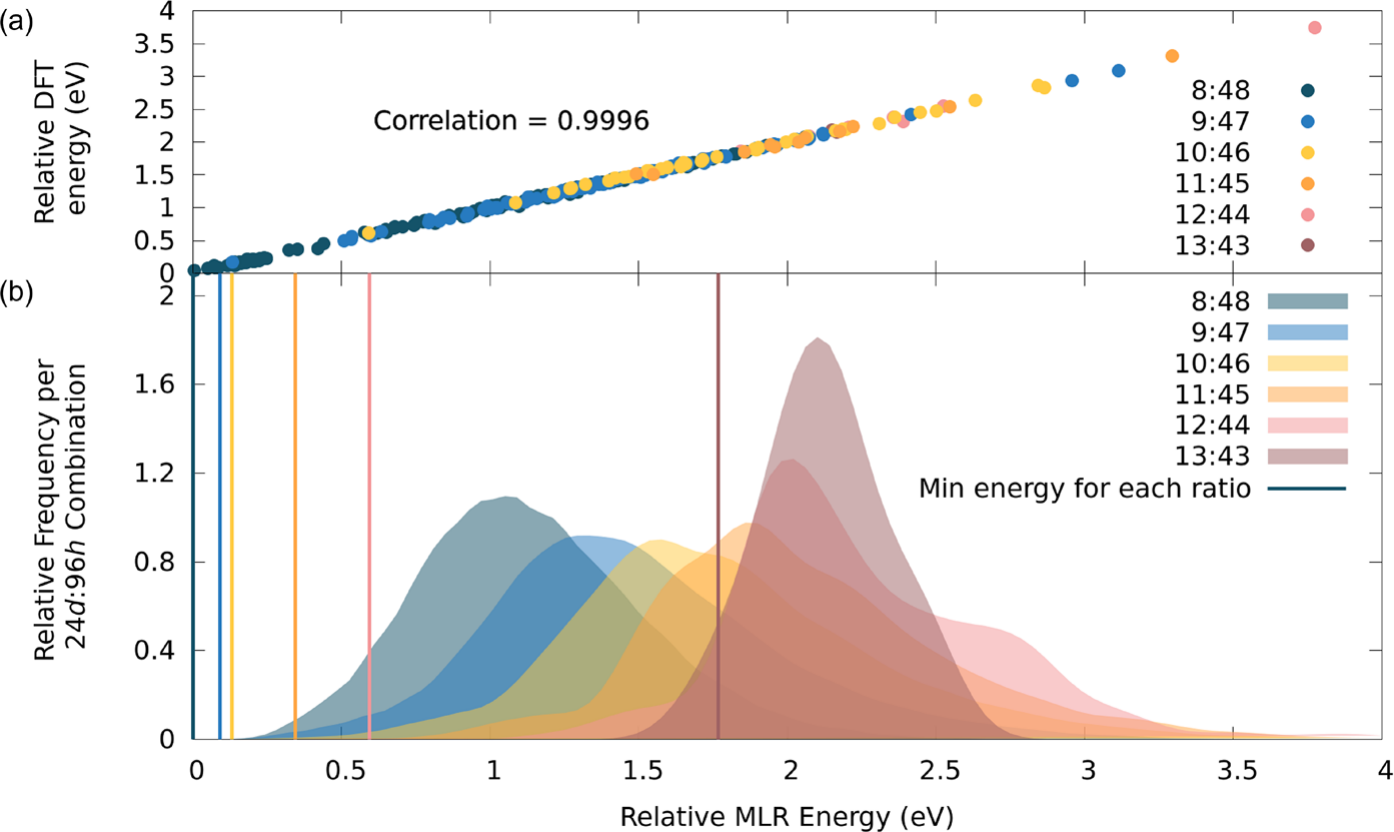
(a) Parity plot of the test set of the ONETEP total energies compared to the MLR-predicted energies for each 24*d*:96*h* ratio. (b) The relative frequency of energy occurrences for each 24*d*:96*h* ratio. Configurational entropic contributions are not included in the energies here.

All structures were found to occur within a range of 5.7 eV from our lowest-energy structure. Seventeen of the 20 lowest-energy structures had a ratio of 8:48 (Figure 2b), while the 17th, 19th, and 20th lowest-energy structures had a ratio of 9:47. The average energy for all of the structures occurred at an energy that was 1.47 eV higher than the ground-state structures. The average energy for each ratio increased with increasing 24*d* occupancy, indicating an energetic preference to avoid 24*d* occupation where possible. There were five structures, all 8:48, within 0.026 eV (1 *kT* at 298 K) of the lowest-energy structure (Figure 3). The five lowest-energy structures have very similar atomic coordinates, all having the same 24*d* configuration with only slight variations in the 96*h* configuration, except for the fourth lowest-energy structure, which has different 24*d* and 96*h* structures. We performed geometry optimization calculations on the final five structures and found that the energy gap between them narrowed further.

**Figure 3:**
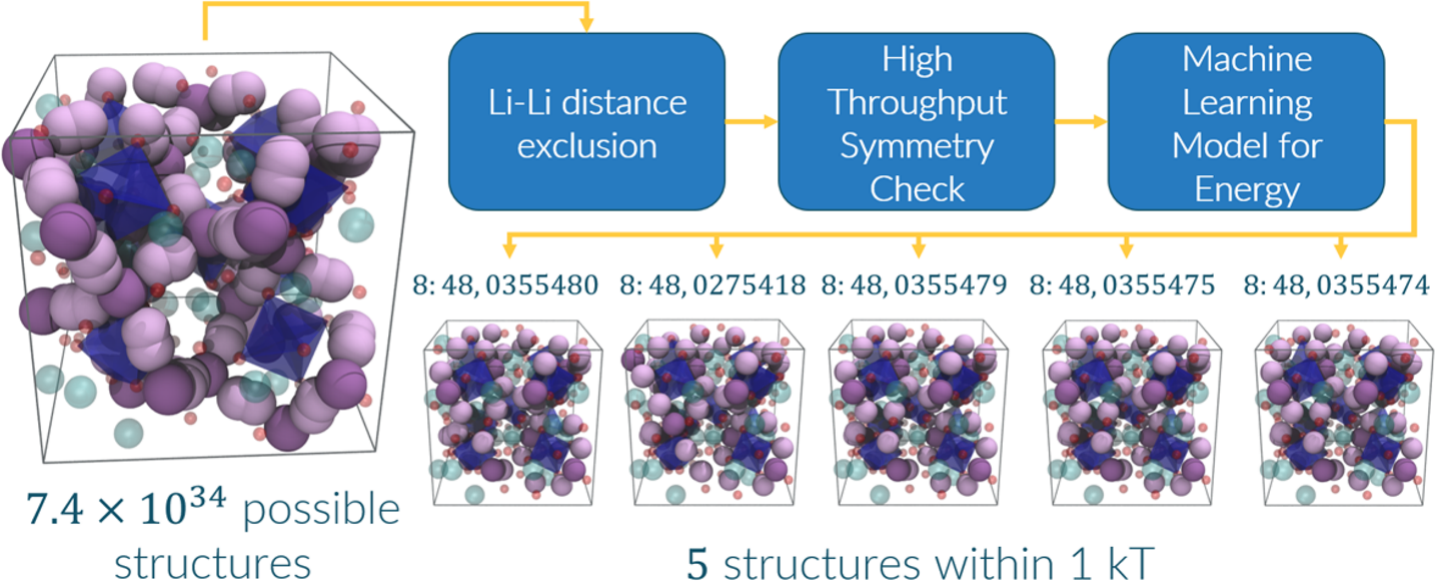
Workflow we employed to find our five final structures from the given occupancies. Above each structure is its respective 24*d*:96*h* ratio and unique identity number in the *c*-LLZO database we have built (see Section S1 of the SI).

We note that our energetic ordering procedure does not include vibrational entropic contributions and assumes a reasonable retention of ordering upon geometry relaxation, which are both approximations. We acknowledge the necessity of finding a good energetic ordering for the optimized structure of *c*-LLZO and have made preliminary efforts toward that. Specificallly, we tested the effect of geometry relaxation on a small data set of 20 structures (SI, Section S12). However, a large-scale energy prediction of the geometry optimized structures requires considerable computational effort and therefore falls outside the scope of this Letter.

It should be stressed that pure *c*-LLZO is not stable at room temperature and requires dopants (typically Al or Ga on the 24*d* sites).^56^ That being said, the structures we provide alongside this work should give an excellent starting point for all future studies on dopants. The addition of dopants will significantly increase the complexity of this problem. Before attempting such a feat, though, we believe that understanding the potential energy surface of the geometry optimized structures is a more pressing matter.

In summary, we created a fast evaluation procedure to generate and energetically order all crystallographically predicted structures for crystals with partially occupied sites. We then used the basis of this procedure, disallowing structures with atoms too close to each other, to highlight that a large proportion of experimental and theoretical literature are predicting or working with structures that are not reflective of a real system (Figure 1). It is our hope that, in providing all possible structures, we can bring further accuracy and reproducibility to future computational LLZO research.

## Supporting Information Available

Access to the database of this work, tables summarizing the experimental and theoretical literature studies mentioned in this work, a Li spacing graph, an energy versus Li–Li distance graph, and details regarding the reduction of configuration space, alternative energy calculations, ONETEP calculations, MLR energy calculations, the symmetry of structures, configurational entropy, and geometry optimizations of the structures (PDF)
Transparent Peer Review report available (PDF)
