# Supplementary material for: A Workflow for Identifying Viable Crystal Structures with Partially Occupied Sites Applied to the Solid Electrolyte Cubic Li7La3Zr2O12
Source: J Phys Chem Lett. 2023 Nov 8;14(45):10257–62. doi: 10.1021/acs.jpclett.3c02064 (PMC10686666; doi:10.1021/acs.jpclett.3c02064)
Supplement: Supplementary file 1 — jz3c02064_si_001.pdf [file jz3c02064_si_001.pdf]

Supplemetary Information: A Workflow for Identifying Viable Crystal Structures with Partially Occupied Sites Applied to Solid Electrolyte: Cubic $Li_7La_3Zr_2O_{12}$

Julian Holland[1,3], Tom Demeyere1, Arihant Bhandari[1,3], Victor Milman[2], Felix Hanke[2] and Chris-Kriton Skylaris[1,3]

[1]School of Chemistry, University of Southampton, Southampton SO17 1BJ, UK
[2]BIOVIA, Unit 334 Cambridge Science Park, Milton Road, Cambridge, Cambridgeshire, CB4 0WN, UK
[3]The Faraday Institution, Quad One, Becquerel Avenue, Harwell Campus, Didcot, OX11 0RA, UK

Contents

S1 Data **2**

S2 Experimental Literature Summary Table **2**

S3 Theoretical Literature Summary Table **3**

S4 Full Region of sufficient Li spacing **4**

S5 Reduction of configuration space **4**

S6 Alternative Energy Calculators **4**

S7 ONETEP details **6**

S8 Multiple Linear Regression Energy Calculator **6**

S9 Li Dimer in Vacuum **8**

S10 Symmetry **9**
S10.1Symmetry Checking 9
S10.2Symmetry Against Energy 9

S11 Configurarional Entropy **9**

S12 Geometry Optimisations **10**

Supplementary Material

S1 Data

All structures, ONETEP input and output files, and codes used in this work can be accessed at https://doi.org/10.5258/SOTON/D2704

S2 Experimental Literature Summary Table

Table S1: All Experimental papers we could find that explicitly report the site occupancies of the $24d$ and $96h$ sites. The second column shows the paper and the reported stoichiometry. The third column reports the method used with "X-ray" referring to some form of X-ray diffraction and "Neutron" referring to any presence of Neutron powder diffraction being present in the assignment. The remaining columns are taken directly from the respective papers and standardised to report the proportion of sites occupied out of total sites and the number of atoms that would result in a full cubic structure
* Ga occupies some of the La $24c$ sites. We have also approximated the $12a$ and $12b$ sites to be the same as the $24d$ sites, as well as the two $48e$ Li sites to be $96h$ so we can compare with the other structures.
† a significant portion of Li reported was unassigned

	Paper<br>Assigned Stoichiometry	Method	Li $24d$ Fraction (Occupancy)	Dopant $24d$ Fraction (Occupancy)	Li $96h$ Fraction (Occupancy)	Total Assigned Sites Occupied
**a**	Xie et al. [1]<br>$Li_7La_3Zr_2O_{12}$	X-ray	0.564 (13.53)	-	0.442 (42.43)	55.96
**b**	Geiger et al. [2]<br>$Li_{4.47}La_3Zr_2O_{12}$	X-ray	0.37 (8.88)	-	0.28 (26.88)	35.76
**c**	Awaka et al. [3]<br>$Li_7La_3Zr_2O_{12}$	X-ray	0.94 (22.56)	-	0.349 (33.504)	56.08
**d**	Brugge et al. [4]<br>$Li_{6.6}Ge_{0.1}La_3Zr_2O_{12}$	Neutron	0.943 (22.64)	Ge 0.033 (0.8)	0.315 (30.24)	53.68
**e**	Buschmann et al. [5]<br>$Li_{6.06}Al_{0.2}La_3Zr_2O_{12}$	Neutron	0.54 (12.96)	Al 0.0653 (1.44)	0.37 (35.52)	49.92
**f**	Hiebl et al. [6]<br>$Li_7Al_{0.33}La_3Zr_2O_{12}$	X-ray	0.712 (17.08)	Al 0.11 (2.64)	0.406 (38.976)	58.696
**g**	Wagner et al. [7]<br>$Li_{6.03}Al_{0.08}La_3Zr_2O_{12}$	X-ray	0.68 (16.32)	Al 0.076 (1.824)	0.32 (30.72)	48.864
**h**	Rettenwander et al. [8†]<br>$Li_{5.5}Al_{0.19}La_3Zr_2O_{12}$	Neutron	0.54 (12.848)	Al 0.064 (1.528)	0.31 (30.0)	44.376
**i**	Rettenwander et al. [8†]<br>$Li_{5.5}Al_{0.20}Ga_{0.05}La_3Zr_2O_{12}$	Neutron	0.57 (13.65)	Al 0.066 (1.584),<br>Ga 0.016 (0.384)	0.31 (29.344)	44.96
**j**	Rettenwander et al. [8†]<br>$Li_{6.3}Al_{0.12}Ga_{0.08}La_3Zr_2O_{12}$	Neutron	0.67 (16.08)	Al 0.039 (0.944),<br>Ga 0.027 (0.64)	0.35 (33.752)	51.416
**k**	Robben et al. [9*]<br>$Li_{6.25}Ga_{0.52}La_{2.67}Zr_2O_{12}$	X-ray	0.811 (19.464)	Ga 0.063 (1.512)	0.315 (31.68)	52.656
**l**	Howard et al. [10]<br>$Li_{5.5}Ga_{0.5}La_3Zr_2O_{12}$	Neutron	0.263 (6.312)	Ga 0.167 (4.008)	0.393 (37.728)	48.048
**m**	Wagner et al. [7]<br>$Li_{6.58}Ga_{0.016}La_3Zr_2O_{12}$	X-ray	0.984 (23.616)	Ga 0.016 (0.384)	0.30 (28.8)	52.8

S3 Theoretical Literature Summary Table

Table S2: All theoretical studies we could find who explicitly report their $24d$:$96h$ ratio and a brief summary of how their structures were generated. If a dopant is present it is included in the total $24d$:$96h$ ratio. We should note that under the majority of these schemes the $24d$:$96h$ ratio is likely not maintained after allowing their structure to relax or move but the final ratio is rarely reported.
† Methodology is discussed further in text

	Paper	Li $24d$:$96h$ Ratio	Method
**n**	Tian et al.[11]	13:43	Use experimentally defined ratio[1] and generate 10 random structures
**n**	Gao et al.[12]	13:43	Use experimentally defined ratio[1] and generate 10 random structures
**n**	Yu et al.[13]	13:43	Unpublished electrostatic minimisation method
**n**	Thompson et al.[14]	13:43	Unpublished electrostatic minimisation method
**n**	Sharafi et al.[15]	13:43	unpublished electrostatic minimisation method
**n**	Sharafi et al.[16]	13:43	Unpublished electrostatic minimisation method
**n**	Yu and Siegel[17]	13:43	perform a quasi-random distribution of Li while keeping the occupancy of sites set
**n**	Barai et al.[18]	13:43	quasi-random distribution
**n**	Rettenwander et al.[19]	13:43 (Al)	Highest symmetry possible (for computational costs)
**o**	Zhang et al.[20]	11:37	Use experimentally defined ratio[3]* and geometry relaxes structure. Explanation is not given as to why the total number of Li changes
**p**	Xu et al.[21]	24:32	Fill all $24d$ sites, randomly distribute the $96h$ then perform a geometry relaxation
**p**	Karasulu et al.[22]	24:32	Created fully lithiated cell and reduced the symmetry to the $R\bar{3}c$ space group then sequentially introduced vacancies
**q**	Jalem et al.[23]	14.64:40.05	AIMD at 1800 K
**r**	Meier et al.[24]	23:33	120 randomly distributed structures with ratio given by experiment[3]
**s**	Santosh et al.[25]	22.56:32.64	Started with experimental results[3] and optimised
**t**	Verduzco et al.[26]	10.59:45.51	Average occupancies after 25 ps of AIMD at 1273 and 1773 K
**u**	Verduzco et al.[26]	11.05:44.60	Average occupancies after 25 ps of AIMD at 1273 and 1773 K
**v**	Haarmann et al.[27]	8:48 (Al)	Combinatorial Software + AIMD†

Haarmann et al.[27] perform the most rigorous exploration of c-LLZO we found. They use the generalised combinatorial structure generation software, Supercell,[28] to generate a number of possible structures. However, due to the large number of structures in a typical c-LLZO cell, let alone a 2x2x2 supercell with an Al present, they limited the configuration space by only placing 48 Li exclusively at $48g$ sites and relying ab-initio molecular dynamics (AIMD) and geometry relaxation to move the Li into the correct 96h site. They allow the remaining Al and Li atoms the freedom of the 24d sites and generate all combinations and select the supercell with the lowest Coulombic energy to proceed with. There are 2 issues with this procedure. Firstly, the choice of the initial structure was mostly for the convenience of a smaller phase space and a low-energy structure is not necessarily guaranteed even with AIMD. Secondly, as we show in our supporting information, the Coulombic forces alone is not sufficient to energetically order c-LLZO structures.

S4 Full Region of sufficient Li spacing

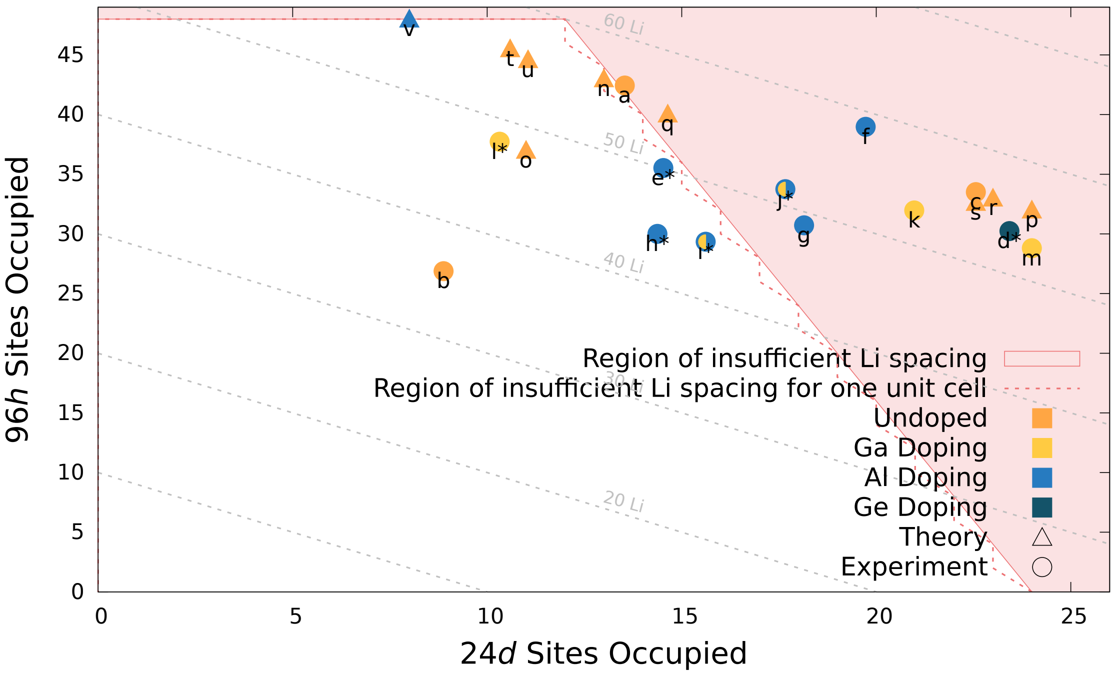

Figure S1: Zoomed out version of figure 1c showing the full area defined by equations 1 and 2.

S5 Reduction of configuration space

The total number of structures (x) available for each 24d:96h ratio (i) for a single unit cell of a given Li occupancy (N), 56 for the stoichiometric case, can be calculated as

$$x_i = \binom{24}{i} + \binom{120 - 4i}{N - i} \tag{1}$$

For a stoichiometric unit cell we can calculate the total number of structures (x_{tot}) as

$$x_{\text{tot}} = \sum_{i=8}^{13} \binom{24}{i} + \binom{120 - 4i}{56 - i} \approx 1.93 \times 10^{25} \tag{2}$$

S6 Alternative Energy Calculators

When attempting to energetically order all 2×10^6 structures we initially attempted to use already available methods such as force fields, which neglect explicitly modelling the electrons in favour of fitted potentials, or Density functional tight-binding (DFTB) which is based on a second-order expansion of the Kohn-Sham total energy functional.[29] These attempts were unsuccessful as they were unable to reproduce the energetic ordering produced by a sample DFT calculation batch. A comparison of the individual methods can be seen in figure S2.

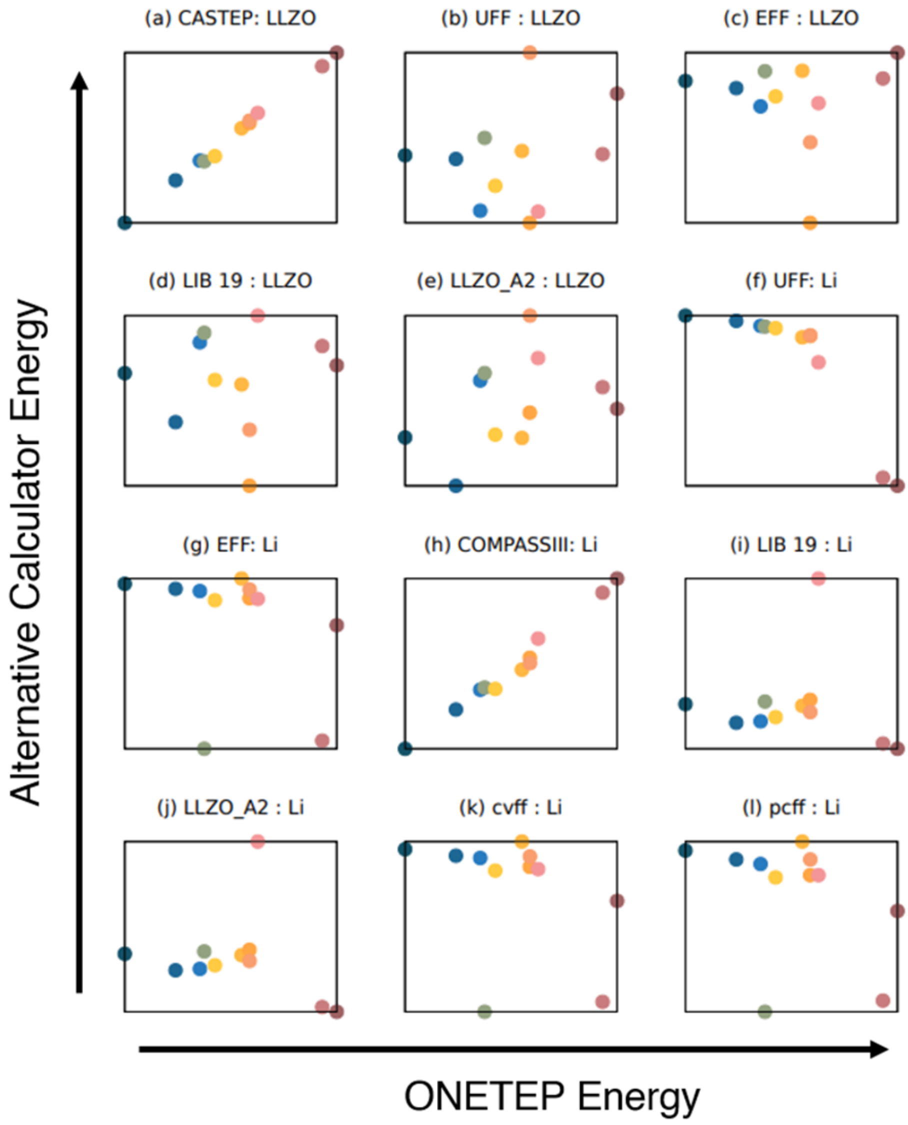

Figure S2: A comparison of the energetic ordering of all symmetry unique 13:43 structures for different energy calculators compared to our ONETEP calculations: (a) CASTEP:[30] DFT software used as our baseline comparison, (b) and (f) Forcite:[31] Universal forcefield,[32] (c) and (g) Forcite:[31] custom forcefield that exclusively considers the Coulombic interaction of atoms, (d) and (i) DFTB+:[33] Slater-Koster library developed for Li-ion batteries[31] and extended to include La and Zr interactions (if required) with data from the LLZO_A2 Slater-Koster library, (e) and (j) DFTB+:[33] unpublished custom parameterised Slater-Koster file specifically for LLZO, (h) Forcite:[31] using the COMPASSIII forcefield,[34] (k) Forcite:[31] using the cvff forcefield,[35] (l) Forcite:[31] using the pcff forcefield[36]
Orderings with "Li" in the title indicate that only the Li sublattice was used in the energy evaluation. "LLZO" indicates all atoms were used. Energy values are not included as they often (in the case of the Li-only calculations) do not correspond to real systems. We are only interested in the ordering.

Due to the limited number of forcefields available with La and Zr parameters and that we predict the majority of the energy differences to come from the unique Li configurations we believed that excluding non-Li elements may still be sufficient to energetically order our structures. Pure electrostatic summation does not appear to be sufficient in this case (c.f. figure S2(g)), which is why additional non-bonded terms such as van der Waals interactions appear necessary. The most successful method we found was COMPASSIII when applied only to the Li sublattice. It was able to order the 13:43 with a correlation coefficient of 0.991.

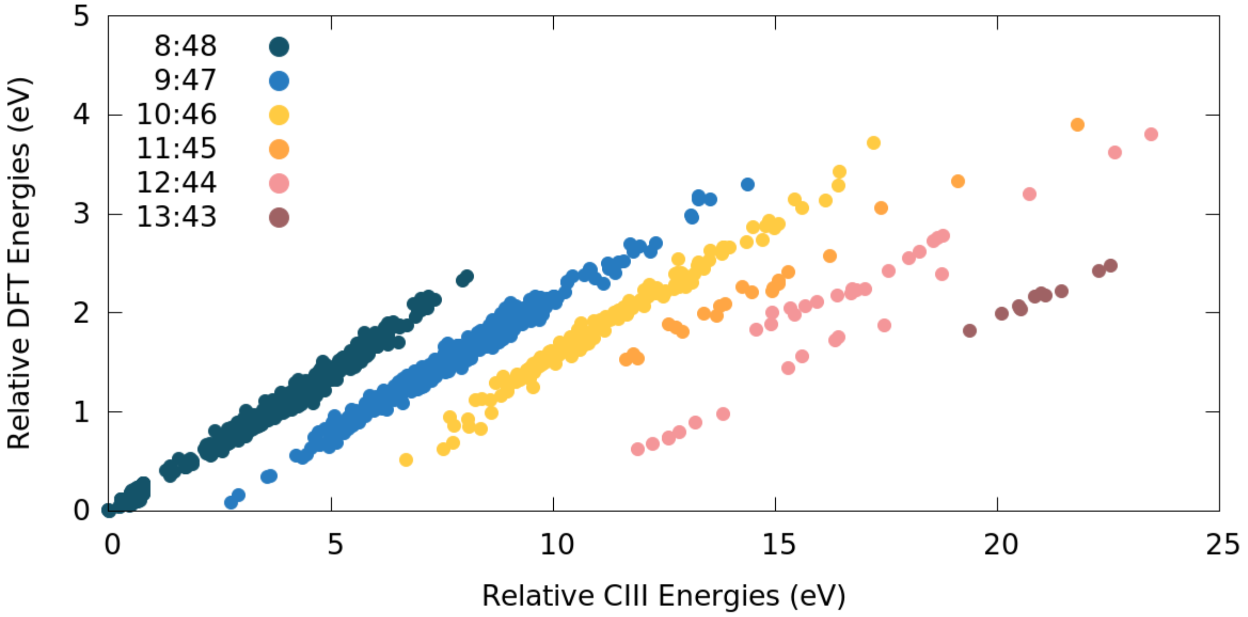

Figure S3: A comparison of 1235 relative DFT energies (found with settings described in SI section S7) to that of COMPASSIII

Figure S3 shows that COMPASSIII, when only evaluated for Li atoms, predicts a more pronounced separation of energies depending on the $24d$:$96h$ ratio than is predicted by ONETEP, while good intra-ratio ordering is retained. This is the best we could do with easily available force fields, but ultimately insufficient for structural ordering. For this reason, we developed the multiple linear regression model. No results in the main part of the paper are actually built on COMPASSIII.

S7 ONETEP details

The following settings were used for our ONETEP calculations:

- Functional: PBE
- Kinetic Energy Cut-off: 830 eV
- Pseudopotentials: CASTEP on-the-fly generated norm-conserving pseudopotentials [30]
- NGWF Radii:
  - Li: 9.0 bohr
  - La: 10.0 bohr
  - Zr: 10.0 bohr
  - O: 9.0 bohr
- NGWF number:
  - Li: 5
  - La: 17
  - Zr: 10
  - O: 5

S8 Multiple Linear Regression Energy Calculator

The multiple linear regression scheme works on the basis of finding the best linear fit using the residual sum of squares (RSS)[37] for a given set of input variables x_i, which form a 1-dimensional vector, to predict an output variable y. The relationship that the vast majority of computational research wishes to establish is one between the atomic positions of

a system and its energy. Often it is the case that a linear relationship cannot be established, or at least the number of required input parameters to find a general scheme is too large and complex to effectively define.

However, when we constrain the total atomic subspace down to a group of very similar systems that are dependent on easily defined variables (such as the occupancy of predefined sites) then a multidimensional linear relationship between a geometric description and energy can be made.

For our system, to reduce the required information for each structure to a vector while carrying as much information as we perform the routine summarised in figure S4

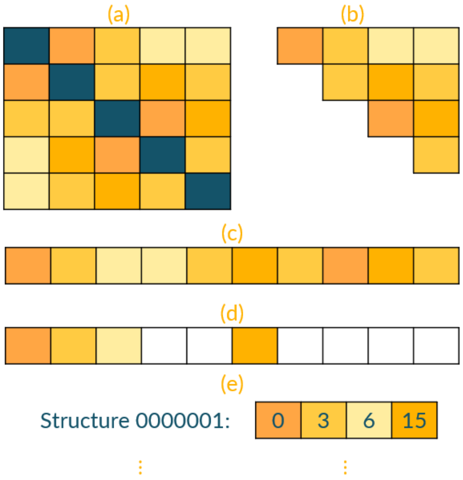

Figure S4: the routine we perform to convert the 3-dimensional coordinates of a string of numbers to a single vector that retains enough information about the system to be predictive. We use a 5×5 Li sublattice as an example here. (a) represents the distance matrix of the periodic Li sublattice, (b) is the upper triangular matrix of a, (c) is the flattened version of b, (d) is all the unique values of c, and (e) is a tally of the number of times this distance occurs in a real, disordered crystal

This routine gives, by necessity, a unique vector for each structure. We find for 120 Li sites there are 86 types of possible Li-Li distance. This means each structure can be represented as a vector of 86 length where each number in that vector represents the number of Li-Li interactions occurring

Having prepared the data, we can now turn to training our model. We chose to run DFT calculations on two separate types of data sets, one set focuses on the low energy structures as predicted by COMPASSIII (c.f. section S6) which are only 8:48. This ensures there is proper sampling over small deviations in the input vectors. The second set, which is larger, are random samples across all of the configuration space we generate this ensures the model has encountered a suitable breadth of structure types. Finally, we can split the test and train set with a ratio of 1:4. The MLR model then creates a linear fit in 86 dimensions to the training data. We show in the main text that when applied to the test set the model is able to reproduce DFT energies with a Pearson coefficient of 0.9996.

We provide examples of how the increasing dimensions of an MLR fit greatly improve the predictive power of the model in figure S5

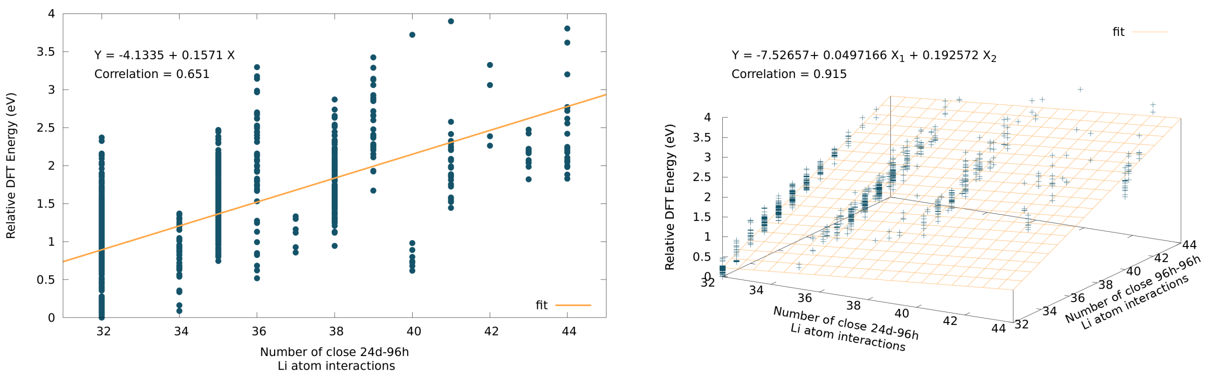

(a) (b)

Figure S5: How increasing the dimension of the input variable with relevant input parameters can greatly improve the predictive power of an MLR model. The yellow line/grid represents the MLR fit in both. (a) is an MLR fit with only one Li-Li interaction tally variable, (b) is an MLR fit with two Li-Li interaction tally variables

We also acknowledge no feature engineering was performed here. This meant we did not remove highly correlated or constant input values, we also didn't normalise our values so that every interaction had an equal weighting. However, given the impressive predictive power of our current model, we found these steps to be unnecessary to achieve the desired results but would recommend implementing them for anyone trying to improve upon our work.

S9 Li Dimer in Vacuum

To find the optimal inter-atomic distance of a Li-dimer we performed 30 single-point calculations in a large unit cell to prevent self-interaction. We use the same, relevant, settings as used in SI section S7. These calculations were performed to generate the 'Li Dimer' quantity in figure 1d as well as to demonstrate the relative energy penalty for bringing Li atoms as close together as 1.7 Å

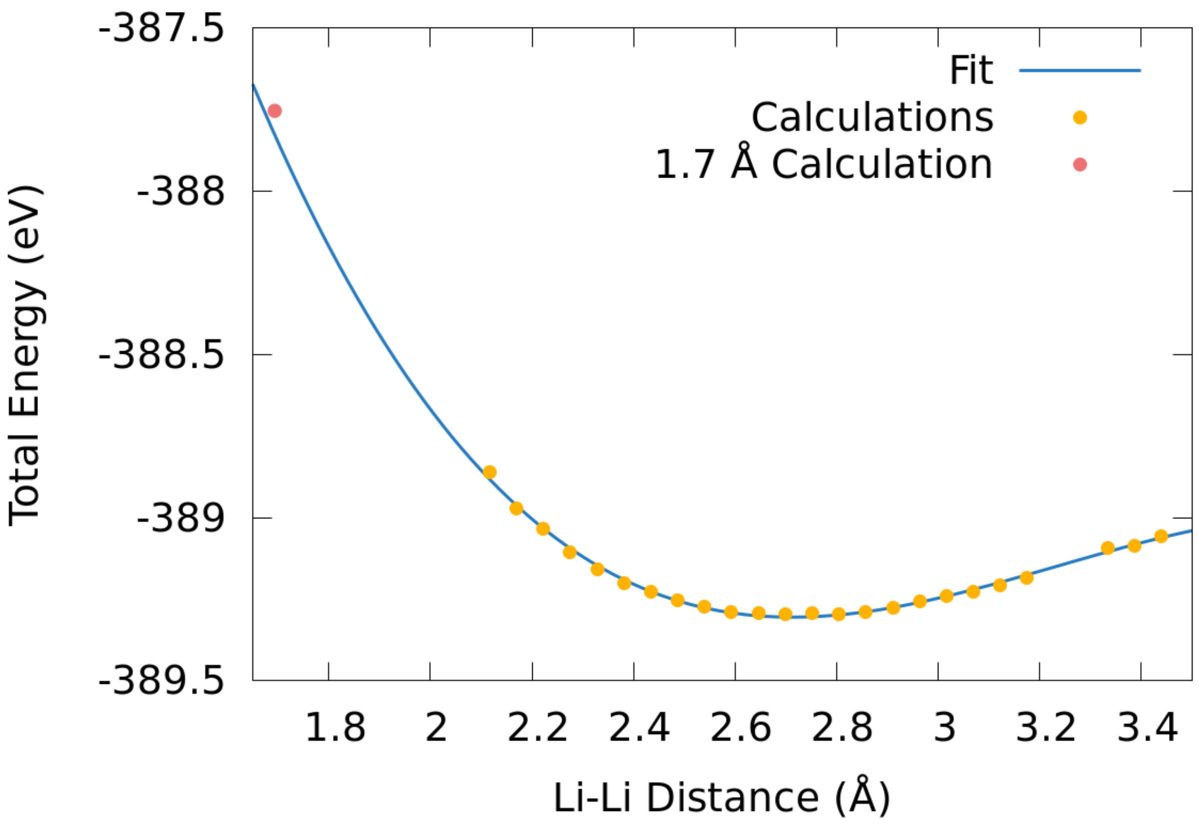

Figure S6: The energy of a Li Dimer with changing inter-atomic distance, the fit is a 3rd order polynomial of the calculations close to the optimal distance (yellow). the 1.7 Å calclulation (red) is displayed for comparison.

Using the fit in figure S6 we find the minimum energy likely to occur at approximately 2.7 Å. We also observe the large energy penalty for Li atoms approaching 1.7 Å of each other.

S10 Symmetry

S10.1 Symmetry Checking

To validate our symmetry checking methodology we compare our results against those of more rigorous methods.[38] We use the atomic simulation environment (ASE) python package's symmetry equivalence check on the two smallest $24d$:$96h$ ratios (c.f. table 1): 12:44 and 13:43.

In both cases, the same number of structures were produced using either method. Both scripts are provided with our database in SI section S1.

S10.2 Symmetry Against Energy

Each generated c-LLZO structure will have a symmetry that is a reduced version of the $Ia\bar{3}d$ space group of the general crystal. We have assigned the new space groups for all 2×10^6 symmetry unique structures, knowing the group we can plot the order of the group against the predicted multiple linear regression (MLR) energy, where the order is the number of unique symmetry operations belonging to an individual space group. Configurational entropy has not been considered here.

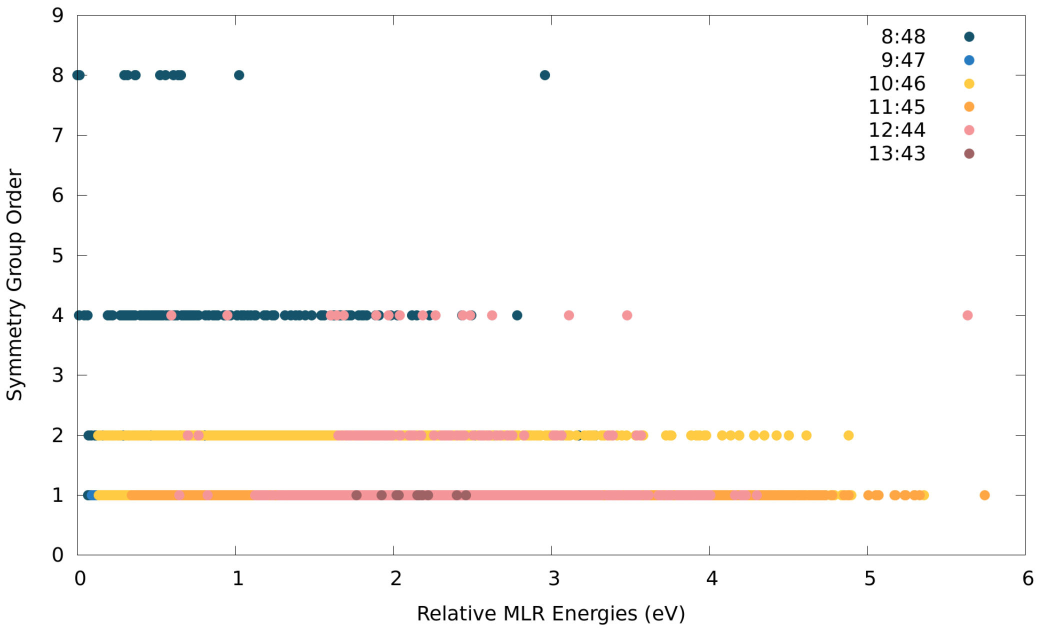

Figure S7: MLR energies compared to the order of the individual structure space group, for each $24d$:$96h$

The most symmetric ratio appears to be 8:48 followed by 12:44 and finally 10:46 with very few odd-numbered $24d$ occupancies having more symmetry operations higher than 1 (the identity). there does appear to be a slight correlation between lower energy and higher symmetry.

We tested including the symmetry of the structure in our MLR model but found it made a negligible difference to the overall results.

S11 Configurarional Entropy

The number of configurations that contribute to configurational entropy for each individual structure can be calculated as the number of symmetry operations (the order) belonging to the $Ia\bar{3}d$ (96) space group which is then divided by the order of the distributed Li structures.

$$\frac{O_{Ia\bar{3}d}}{O_{\text{Li Sublattice}}} = \Omega \tag{3}$$

We can then use this number in the following equation to get the configurational entropy, S_{config}

$$-TS_{\text{config}} = -k_B T \ln(\Omega) \tag{4}$$

Where T is the temperature and k_B is the Boltzmann constant. The vast majority of structures do not have any symmetry operations other than the identity so belong to the space group $P1$. However, some structures have more than the identity operator available to them and will therefore have different entropic contributions. For this reason, the configurational entropy will need to be considered when energetically ordering our structures. We find structures with space group orders of 1, 2, 4, and 8 (c.f. figure S7).

Table S3: All space group orders we recorded and their configurational entropic contributions at 298.15 K

Space group order of the Li sublattice	Number of occurrences	Configurational Entropy Contribution at Room Temperature (eV)
1	2154949	-0.1173
2	3635	-0.09946
4	174	-0.08165
8	14	-0.06384

Table S3 shows that the maximum energy difference that can occur due to configurational entropy is 53 meV. We can also see that only 0.18% of the structures are going to have a different S_{config} than the majority, therefore ordering will be minimally affected.

S12 Geometry Optimisations

To assess what effect the geometry relaxed structures have on the overall ordering and also to compare our low energy structures to that of a random selection we perform 20 geometry optimisation calculations. We use the settings outlined in SI section S7 as well as the following geometry optimisation settings for the Broyder-Fletcher-Goldfarb-Shanno (BFGS) [39] optimisation algorithm

- Energy tolerance: 1.0E-6 hartree
- Force tolerance: 0.002 ha/bohr
- Displacement tolerance: 0.005 bohr

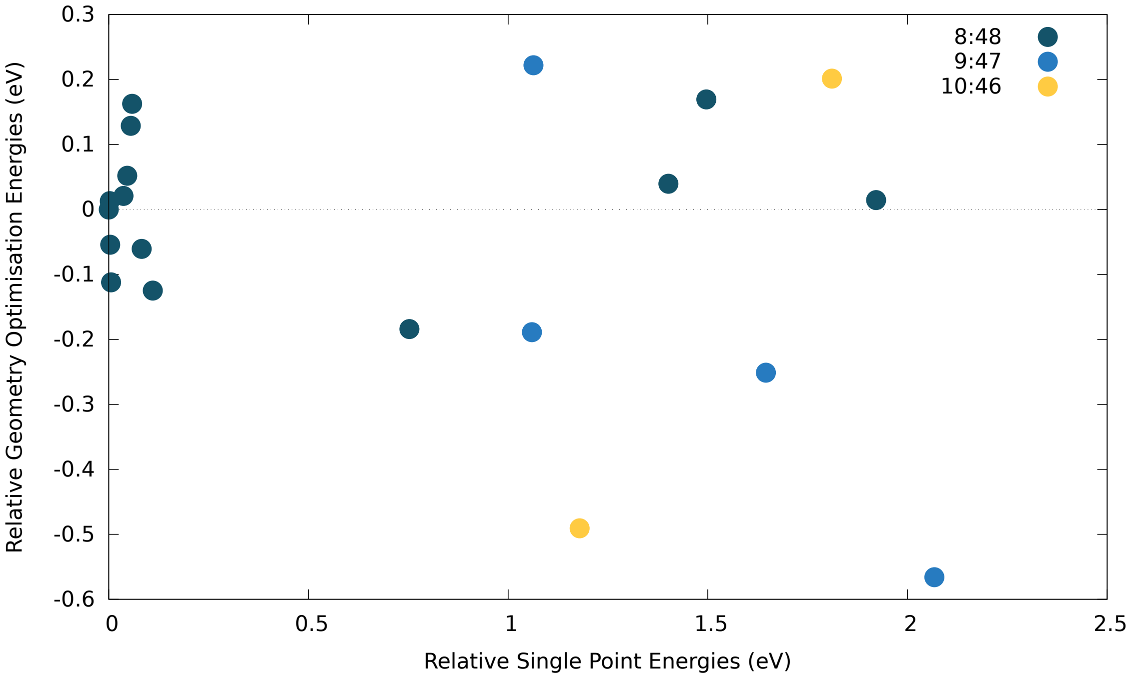

Figure S8: A comparison of the energetic ordering of 20 structures (10 lowest structures according to MLR and 10 random structures). Single point and geometry optimisation calculations were performed using DFT for all structures. All energies are relative to the lowest MLR energy structure.

Figure S8 demonstrates no correlation between the single-point structures and their geometry optimised counterparts. We observe three types of site change upon relaxation. $96h$ to $96h$, $96h$ to $48g$ and $96h$ to $24d$ listed in order of frequency occurred. None of these site changes correlated significantly with the geometry optimised energy. The $96h$ to $24d$ transition was only recorded once and yielded our lowest overall energy structure (structure label 1095859): 0.58 eV lower in energy than the geometry optimised lowest energy single point structure. The $96h$ to $24d$ transition meant that the ratio of the structure changed from 9:47 to 10:46, the only time we have observed such a feature.

In figure S9 there is a negative correlation between the mean displacement of all Li atoms in the c-LLZO structure as a result of the geometry-optimisation and the energy of the final optimised c-LLZO structure. This indicates that the more the Li atoms move from their original crystallographic site the lower the energy of the structure. Figure S9b shows a weaker negative correlation between distance to a crystallographic site and energy. These results indicate that from the structures assessed movement from the original sites is preferable to attain lower energies and movement to another site is not necessary in order to attain a lower energy structure.

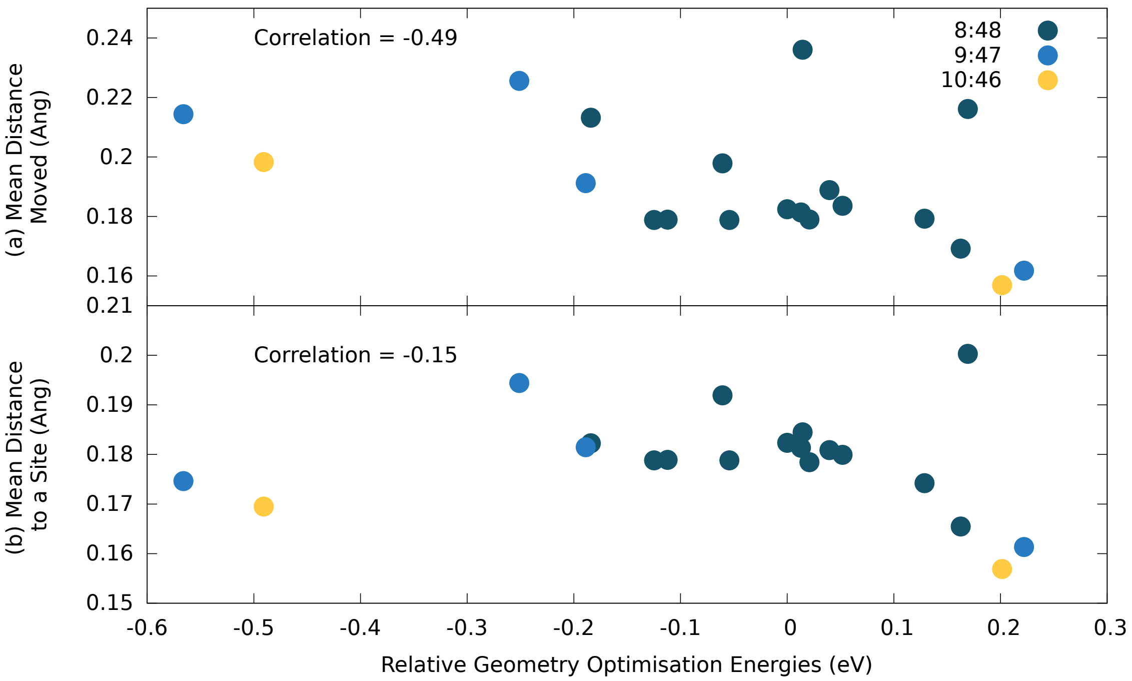

Figure S9: The relationships of the energy of 20 geometry optimised c-LLZO structures with (a) the mean distance moved by the individual Li atoms from their original crystallographically predicted site and (b) the mean distance of all Li atoms from a crystallographically identified Li site. Colours identify the $24d$:$96h$ ratio of the starting structure.

References

[1] Xie, H.; Alonso, J. A.; Li, Y.; Fernández-Díaz, M. T.; Goodenough, J. B. *Chemistry of Materials* **2011**, *23*, 3587–3589.

[2] Geiger, C. A.; Alekseev, E.; Lazic, B.; Fisch, M.; Armbruster, T.; Langner, R.; Fechtelkord, M.; Kim, N.; Pettke, T.; Weppner, W. *Inorganic Chemistry* **2011**, *50*, 1089–1097.

[3] Awaka, J.; Takashima, A.; Kataoka, K.; Kijima, N.; Idemoto, Y.; Akimoto, J. *Chemistry Letters* **2011**, *40*, 60–62.

[4] Brugge, R. H.; Kilner, J. A.; Aguadero, A. *Solid State Ionics* **2019**, *337*, 154–160.

[5] Buschmann, H.; Dölle, J.; Berendts, S.; Kuhn, A.; Bottke, P.; Wilkening, M.; Heitjans, P.; Senyshyn, A.; Ehrenberg, H.; Lotnyk, A.; Duppel, V.; Kienle, L.; Janek, J. *Physical Chemistry Chemical Physics* **2011**, *13*, 19378–19392.

[6] Hiebl, C.; Young, D.; Wagner, R.; Wilkening, H. M.; Redhammer, G. J.; Rettenwander, D. *Journal of Physical Chemistry C* **2019**, *123*, 1094–1098.

[7] Wagner, R.; Redhammer, G. J.; Rettenwander, D.; Senyshyn, A.; Schmidt, W.; Wilkening, M.; Amthauer, G. *Chemistry of Materials* **2016**, *28*, 1861–1871.

[8] Rettenwander, D.; Redhammer, G.; Preishuber-Pflügl, F.; Cheng, L.; Miara, L.; Wagner, R.; Welzl, A.; Suard, E.; Doeff, M. M.; Wilkening, M.; Fleig, J.; Amthauer, G. *Chemistry of Materials* **2016**, *28*, 2384–2392.

[9] Robben, L.; Merzlyakova, E.; Heitjans, P.; Gesing, T. M. *Acta Crystallographica Section E: Crystallographic Communications* **2016**, *72*, 287–289.

[10] Howard, M. A.; Clemens, O.; Kendrick, E.; Knight, K. S.; Apperley, D. C.; Anderson, P. A.; Slater, P. R. *Dalton Transactions* **2012**, *41*, 12048–12053.

[11] Tian, H.-K.; Xu, B.; Qi, Y. *Journal of Power Sources* **2018**, *392*, 79–86.

[12] Gao, Y.; Nolan, A. M.; Du, P.; Wu, Y.; Yang, C.; Chen, Q.; Mo, Y.; Bo, S. H. *Chemical Reviews* **2020**, *120*, 5954–6008.

[13] Yu, S.; Schmidt, R. D.; Garcia-Mendez, R.; Herbert, E.; Dudney, N. J.; Wolfenstine, J. B.; Sakamoto, J.; Siegel, D. J. *Chemistry of Materials* **2016**, *28*, 197–206.

[14] Thompson, T.; Yu, S.; Williams, L.; Schmidt, R. D.; Garcia-Mendez, R.; Wolfenstine, J.; Allen, J. L.; Kioupakis, E.; Siegel, D. J.; Sakamoto, J. *ACS Energy Letters* **2017**, *2*, 462–468.

[15] Sharafi, A.; Kazyak, E.; Davis, A. L.; Yu, S.; Thompson, T.; Siegel, D. J.; Dasgupta, N. P.; Sakamoto, J. *Chemistry of Materials* **2017**, *29*, 7961–7968.

[16] Sharafi, A.; Yu, S.; Naguib, M.; Lee, M.; Ma, C.; Meyer, H. M.; Nanda, J.; Chi, M.; Siegel, D. J.; Sakamoto, J. *Journal of Materials Chemistry A* **2017**, *5*, 13475–13487.

[17] Yu, S.; Siegel, D. J. *Chemistry of Materials* **2017**, *29*, 9639–9647.

[18] Barai, P.; Ngo, A. T.; Narayanan, B.; Higa, K.; Curtiss, L. A.; Srinivasan, V. *Journal of The Electrochemical Society* **2020**, *167*, 100537.

[19] Rettenwander, D.; Blaha, P.; Laskowski, R.; Schwarz, K.; Bottke, P.; Wilkening, M.; Geiger, C. A.; Amthauer, G. *Chemistry of Materials* **2014**, *26*, 2617–2623.

[20] Zhang, X.; Liu, W.; Yu, Y. *Solid State Ionics* **2021**, *360*.

[21] Xu, M.; Park, M. S.; Lee, J. M.; Kim, T. Y.; Park, Y. S.; Ma, E. *Physical Review B* **2012**, *85*, 052301.

[22] Karasulu, B.; Emge, S. P.; Groh, M. F.; Grey, C. P.; Morris, A. J. *Journal of the American Chemical Society* **2020**, *142*, 3132–3148.

[23] Jalem, R.; Yamamoto, Y.; Shiiba, H.; Nakayama, M.; Munakata, H.; Kasuga, T.; Kanamura, K. *Chemistry of Materials* **2013**, *25*, 425–430.

[24] Meier, K.; Laino, T.; Curioni, A. *Journal of Physical Chemistry C* **2014**, *118*, 6668–6679.

[25] Santosh, K. C.; Longo, R. C.; Xiong, K.; Cho, K. *Solid State Ionics* **2014**, *261*, 100–105.

[26] Verduzco, J. C.; Marinero, E. E.; Strachan, A. *The Journal of Physical Chemistry C* **2023**, *127*, 10806–10812.

[27] Haarmann, L.; Rohrer, J.; Albe, K. *ACS Applied Materials and Interfaces* **2021**, *13*, 52629–52635.

[28] Okhotnikov, K.; Charpentier, T.; Cadars, S. *Journal of Cheminformatics* **2016**, *8*.

[29] Elstner, M.; Porezag, D.; Jungnickel, G.; Elsner, J.; Haugk, M.; Frauenheim, T. *Physical Review B - Condensed Matter and Materials Physics* **1998**, *58*, 7260–7268.

[30] Clark, S. J.; Segall, M. D.; Pickard, C. J.; Hasnip, P. J.; Probert, M. I.; Refson, K.; Payne, M. C. *Zeitschrift fur Kristallographie* **2005**, *220*, 567–570.

[31] BIOVIA, Dassault Systemes, Materials Studio, 23.1.0.3829, San Diego: Dassault Systemes. 2023.

[32] Rappe, A. K.; Casewit, C. J.; Colwell, K. S.; Goddard, W. A.; Skiff, W. M. *Journal of the American Chemical Society* **1992**, *114*, 10024–10035.

[33] Hourahine, B. et al. *The Journal of Chemical Physics* **2020**, *152*, 124101.

[34] Sun, H. *The Journal of Physical Chemistry B* **1998**, *102*, 7338–7364.

[35] Hagler, A. T.; Huler, E.; Lifson, S. *Journal of the American Chemical Society* **1974**, *96*, 5319–5327.

[36] Sun, H.; Mumby, S. J.; Maple, J. R.; Hagler, A. T. *Journal of the American Chemical Society* **1994**, *116*, 2978–2987.

[37] Hastie, T.; Tibshirani, R.; Friedman, J. *The Elements of Statistical Learning: Data Mining, Inference, and Prediction*, 2nd ed.; Springer, 2009.

[38] Togo, A.; Oba, F.; Tanaka, I. *Physical Review B - Condensed Matter and Materials Physics* **2008**, *78*, 1–9.

[39] Shanno, D. F. *Mathematics of Operations Research* **1978**, *3*, 244–256.
