# Supplementary material for: A Workflow for Identifying Viable Crystal Structures with Partially Occupied Sites Applied to the Solid Electrolyte Cubic Li7La3Zr2O12
Source: J Phys Chem Lett. 2023 Nov 8;14(45):10257–62. doi: 10.1021/acs.jpclett.3c02064 (PMC10686666; doi:10.1021/acs.jpclett.3c02064)
Supplement: Supplementary file 2 — jz3c02064_si_002.pdf [file jz3c02064_si_002.pdf]

jz-2023-02064v.R1

Name: Peer Review Information for "A Workflow for Identifying Viable Crystal Structures with Partially Occupied Sites Applied to Solid Electrolyte: Cubic Li₇La₃Zr₂O₁₂"

First Round of Reviewer Comments

Reviewer: 1

Comments to the Author

In this paper, Authors provide a detailed computational combinatorial procedure to generate representative model structures for cubic LLZO having a disordered crystal that is hard to resolve experimentally using the XRD and NPD methods. Authors present a systematic, detailed and well-documented procedure to generate the c-LLZO structures. Calculations presented here are detailed at a high level, which combined with the datasets provided publicly increases the reproducibility of the results. Presentation of the results are clear and visually appealing and the discussions are easy to follow, and comprehensive yet concise, with minimal formatting/grammar/language errors, and the length is suitable for a letter. The literature has been cited properly except for one key reference missing (see below).

Even though Authors present a systematic study and well-written manuscript, I have some major concerns regarding the novelty of the results presented here. A similar computational approach for generating representative cubic-LLZO structures has been reported previously by Karasulu et al (JACS 2020, 142, 6, 3132–3148). That study presents an automated ‘high-throughput’ approach, which relies on the exclusion of Li sites based on Li-Li distances (also excluding 48g sites), reduction of structures (configurations) based on symmetry equivalence, and enumeration of the different structures based on DFT energies using the corresponding optimized structures (rather than DFT single points as presented here). The only major additional step that stands out in the current study is the fitting of the DFT single-point energies using a multiple linear regression (MLR) procedure to energetically order a large set of structures. Interestingly, Authors has -probably inadvertently- excluded this relevant study by Karasulu et al in their discussions. It would be useful to directly compare those findings with the current ones, and comment on the possible source of major discrepancies (if any).

Another vital point, which is only very briefly mentioned in the current discussions, is the fact that the cubic form of LLZO is not stable at room temperature and that various multi-valent chemical dopants (Al, Ga, Ta, etc.) are needed to stabilize this polymorph. And doping elements within the lattice have been shown to completely change the Li atom content and their distribution within the lattice (considering the aliovalency). Overlooking of this fact can significantly limit the physical relevance of the predicted non-doped c-LLZO structures presented here. Taking this into account, Authors should make some efforts to validate their predicted structures using experimental findings that reveal the local coordination environments (micro structures), e.g. via solid-state NMR, EELS, XANES, etc. As the samples used in the

experimental reports contain stabilizing chemical dopants, Authors will need to extend their structure prediction studies to entail the selected chemical dopants (as was done in Karasulu et al paper).

To consider the current manuscript for publication in The Journal of Physical Chemistry Letters, the authors should make the suggested changes/additions. In view of the previous reports with similar approaches, the Authors also need to clarify the novelty and significance of the physical insights presented here. Otherwise, I would recommend considering the manuscript for a more specialized journal like JPCC.

Some specific points:

- More details regarding the multiple linear regression (MLR) procedure should be provided.

- The exact force-field parameters used in the study (Fig. S2), particularly COMPASSIII (which gives the highest agreement with DFT results). Authors cite the original COMPASSIII reference, which is on carbon materials.

- Not so clear why the calculations on the Li dimer in vacuum have been performed.

- For disordered materials, configurational entropy is a major factor that defines the relative stability of a specific atomic configuration / structure. Authors mention that they have excluded these entropic effects altogether without giving much justification or insights into how the final set of lowest-energy structures will be affected by this exclusion.

- The conclusion given in the abstract “By considering the geometrical constraints that emerge from this methodology we determine that a large portion of previously reported structures may not be feasible or stable.” Is not supported by the current results (considering that they do not contain the chemical dopants, as opposed to the literature reports cited here) and hence this statement is probably not valid.

- The assumption that the LaZrO structure does not change much with the distribution of the LI atoms within the lattice has limited validity, as the LaO8 and ZrO6 environments are shown to be affected by the immediate Li neighbors (Karasulu et al 2020 and references therein)

Some minor points:

- References need to be edited as many typos and formatting errors are visible, particularly ref 30.p. 21: ‘All energies are relative to the 355474 result for each calculation type’ sentence is not clear.

- Fig S6: not so clear which data points correspond to the SP and GO data sets.

- Fig. S5: How is the symmetry group order is defined?

Author's Response to Peer Review Comments:

Authors' Response to Reviews of

A Workflow for Identifying Viable Crystal Structures with Partially Occupied Sites Applied to Solid Electrolyte: Cubic $Li_7La_3Zr_2O_{12}$

Julian Holland, Tom Demeyere, Arihant Bhandari, Felix Hanke, Victor Milman, and Chris-Kriton Skylaris
Journal of Physical Chemistry Letters, `jz-2023-02064v`

RC: ***Reviewers' Comment***, AR: Authors' Response, ☐ Manuscript Text

1. Reviewer #1 Report:

In this paper, Authors provide a detailed computational combinatorial procedure to generate representative model structures for cubic LLZO having a disordered crystal that is hard to resolve experimentally using the XRD and NPD methods. Authors present a systematic, detailed and well-documented procedure to generate the c-LLZO structures. Calculations presented here are detailed at a high level, which combined with the datasets provided publicly increases the reproducibility of the results. Presentation of the results are clear and visually appealing and the discussions are easy to follow, and comprehensive yet concise, with minimal formatting/grammar/language errors, and the length is suitable for a letter. The literature has been cited properly except for one key reference missing (see below).

1.1. Comment #1

RC: ***Even though Authors present a systematic study and well-written manuscript, I have some major concerns regarding the novelty of the results presented here. A similar computational approach for generating representative cubic-LLZO structures has been reported previously by Karasulu et al. (JACS 2020, 142, 6, 3132–3148). That study presents an automated 'high-throughput' approach, which relies on the exclusion of Li sites based on Li-Li distances (also excluding 48g sites), reduction of structures (configurations) based on symmetry equivalence, and enumeration of the different structures based on DFT energies using the corresponding optimized structures (rather than DFT single points as presented here). The only major additional step that stands out in the current study is the fitting of the DFT single-point energies using a multiple linear regression (MLR) procedure to energetically order a large set of structures. Interestingly, Authors has -probably inadvertently- excluded this relevant study by Karasulu et al. in their discussions. It would be useful to directly compare those findings with the current ones, and comment on the possible source of major discrepancies (if any).***

AR: We thank the reviewer for highlighting this article and apologise missing it in our original analysis. We have integrated the results (now Ref. 22) throughout the manuscript, specifically to the relevant table and figure collating the literature data. We could not include the experimental data because while an x-ray diffraction has been performed a Rietveld refinement of the various site occupancies was not available to us with this work.

Specifically, we have added the following to table S2

	Paper	Li 24d:96h Ratio	Method
**p**	Karasulu et al.[22]	24:32	Created fully lithiated cell and reduced the symmetry to the $R\bar{3}c$ space group then sequentially introduced vacancies

as well as adding the reference to figure 1

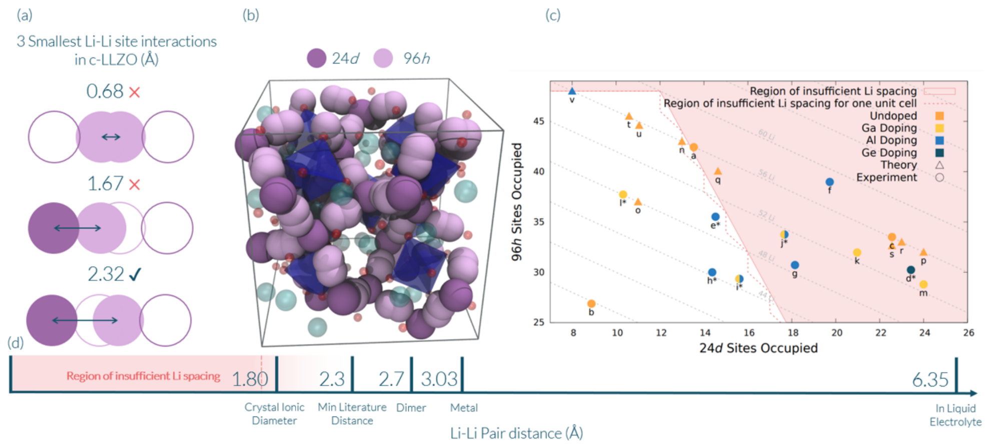

Figure 1: (a) examples of the 3 smallest site interactions from the centre of each atom. First and second neighbour Li sites are too close and cannot simultaneously contain a Li atom. (b) a unit cell of c-LLZO with all 24d (dark purple) and 96h (light purple) sites highlighted. O and La are represented by red and green spheres respectively, the Zr atoms are represented by the blue polyhedra. (c) shows experimental and theoretical site assigments and/or starting structures in the literature: a[1], b[2], c[3], d[4], e[5], f[6], g[7], h[8], i[8], j[8], k[9], l[10], m[7], n[11–15,17–19], o[21], p[23] [22], q[24], r[25], s[26], t[26], u[27]. * indicates that experimental assignments used neutron diffraction, no * means the experimental assignments used x-ray diffraction. The region in red indicates the 24d:96h ratios that cannot exist without a Li-Li interaction below the crystal ionic diameter. The data for this image is provided in the supporting information (Tables S1 and S2). (d) a scale showing the important Li-Li interactions distances recorded in the literature: Crystal Ionic Diameter,[37] Min Literature Distance[38], Metal[39], Dimer (in a vacuum) (c.f. figure S6), In Liquid Electrolyte (average Li-Li distance)[40] as well as our chosen cutoff point at 1.7 Å (red dashed line).

We disagree with the reviewer's claim that this work is largely a repeat of Karasulu et al.'s. There are several key advances in our paper when compared to the one the reviewer mentions in our generation of Li structures. We summarise our method as

1. For all possible 24d occupancies in the Ia-3d space group of c-LLZO generate all possible 24d Li sublattices
2. Remove any 96h sites that correspond to overlapping Li atoms and generate all possible structures from the resulting geometric constraint
3. Symmetry reduce the roughly 200,000,000 structures to 2,000,000

The structure generation method used in the paper the reviewer mentions can be summarised as

1. Start with a fully lithiated c-LLZO structure and reduce the space group to a symmetrically lower R3c
2. Set all 24d sites (Li1) to be fully occupied
3. Sequentially introduce the most energetically favorable vacancy until the Li7LZO stoichiometry is reached
4. 1056 configurations are produced of which the lowest energy (according to a single point calculation) is selected and advanced

We find that our structure accounting technique has has broader applicability for the following reasons.

- We present a straight forward accounting to generate the "allowed" region in Fig 1 that requires knowledge of the connectivity of crystal sites, but nothing else. This technique by itself is already very helpful to guide structure assignment to regions in which no two atoms occupy the same space, a generic requirement that is broadly applicable to many crystal systems beyond LLZO covered here. The methods used to setup Eqn (1) are accessible to all material scientists without requiring deep numerical or computational expertise.
- We worked with the full Ia3d space group which is the reported space group of the experimental literature and not the symmetrically different R3c space group that Karasulu et al. does
- We sample all 24d occupancies that produce structures with allowed Li distances, while the work Karasulu et al. is restricted to a single 24d occupancy which we conclude to Li atoms that appear unphysically close using the method outlined in Eqn (1) and discussed in the previous point.
- We generated over 2 million structures with no assumptions made about the energetically suitability beyond geometric constraints, Karasulu et. al only generated one final structure which was found under the assumption that a sequential removal of Li atoms would yield the most suitable structure, and proceeded with their calculations using that. A slight energy difference at an early stage in their procedure could yield a completely different final structure. We provide all possible structures as a dataset for researchers to make their own investigations with as well as only providing an energetic characterisation as a recommendation instead of a defacto structure.

The reviewer claims that in the Karasulu et. al paper they used geometry optimised structures in their selection criteria. This is true only after they have decided on their c-LLZO structure. Therefore, we do not believe anyone has made a thorough assessment of the configuration space of the optimised structures to date. We acknowledge that single point structures are not ideal at the end of the paper but a necessary constraint due to current methodological and computational restrictions. We expand this section to make it clearer the necessity of this.

> We note that our energetic ordering procedure does not include entropic contributions and assumes a reasonable retention of ordering upon geometry relaxation, which are both approximations. We acknowledge the necessity of finding a good energetic ordering for the optimised structure of c-LLZO and have made preliminary efforts towards that. We have tested the effect of geometry relaxation on a small dataset of 20 structures (c.f. SI section S12). A large-scale energy prediction of the geometry

optimised structures will require considerable computational effort and therefore falls outside the scope of this communication.

1.2. Comment #2

RC: ***Another vital point, which is only very briefly mentioned in the current discussions, is the fact that the cubic form of LLZO is not stable at room temperature and that various multi-valent chemical dopants (Al, Ga, Ta, etc.) are needed to stabilize this polymorph. And doping elements within the lattice have been shown to completely change the Li atom content and their distribution within the lattice (considering the aliovalency). Overlooking of this fact can significantly limit the physical relevance of the predicted non-doped c-LLZO structures presented here. Taking this into account, Authors should make some efforts to validate their predicted structures using experimental findings that reveal the local coordination environments (micro structures), e.g. via solid-state NMR, EELS, XANES, etc. As the samples used in the experimental reports contain stabilizing chemical dopants, Authors will need to extend their structure prediction studies to entail the selected chemical dopants (as was done in Karasulu et al. paper).***

AR: We thank the reviewer for this suggestion and agree that adding a doping and experimental section would be advantageous. However, we believe that such ambitions would be better pursued in a separate piece of literature.

We feel that it is essential to get the c-LLZO structures right before moving on to the complexities of doping, which sevaral studies have struggled to do. Adding dopants is an obvious next step but ultimately beyond the scope of this manuscript. We believe the current manuscript accelerates computational advances in area by providing all physically meaningful c-LLZO structures. We also reiterate the broad applicability of our geometric constraint accounting to other crystal systems.

As the authors of the Karasulu et al. paper point out the dopant range that stabilises the LLZO is very wide $0.07 < a < 0.53$ in $\mathrm{Li}_{7-3a}\mathrm{X}_a$LZO. Which as a minimum is less than one dopant per unit cell thereby making our structures by no means unphysical for a local environment in a bulk c-LLZO crystal. The goal of this paper was to generate the general structure of c-LLZO without dopant so that future researchers can use these structures generated to perform their own investigations, for example by adding dopants.

We have further stressed the importance of the eventual inclusion of dopants in this paper.

> It should be stressed that pure c-LLZO is not stable at room temperature and requires dopants (typically of Al or Ga on the 24*d* sites[56]). However, the structures we provide alongside this work should provide an excellent starting point for all future studies on dopants. The addition of dopants will significantly increase the complexity of this problem. Before attempting such a feat we believe understanding the potential energy surface of the geometry optimised structures to be more pressing.
>
> In summary, ...

1.3. Comment #3

RC: ***To consider the current manuscript for publication in The Journal of Physical Chemistry Letters, the authors should make the suggested changes/additions. In view of the previous reports with similar approaches, the Authors also need to clarify the novelty and significance of the physical insights presented here. Otherwise, I would recommend considering the manuscript for a more specialized journal like JPCC.***

AR: We thank the reviewer for this recommendation and feel we have answered the comments to a standard worthy

of publication in this journal. We have already addressed our unique search methodology that systematically provides all possible structures that can occur outside a provided Li-Li distance in comment 1. We are not aware of existing works that do anything similar.

Given the scale of the configuration space that we produced a new symmetry checking methodology had to be produced. This symmetry checking methodology employed cutting-edge computational science techniques such as local sensitivity hashing to enable a comparison of over 200,000,000 structures. This left us with around 2,000,000 structures.

These 2,000,000 structures are published alongside our work and are guaranteed to be "low-energy" (i.e. no Li close contacts) and encapsulate the entire reasonable configuration space of the c-LLZO structure. This effectively removes the need for future works to generate their own structures. Something, given the variety of different methodologies used in table S1 that has clearly taken up a large amount of time and effort in multiple groups across the field. We energetically resolve the produced structures using a simple machine learning technique that is able to replicate DFT energies with 99.96% accuracy which we provide as a guide for those wanting to use our structures.

Finally, we once again want to highlight the utility of the configurational map in Fig. 1 in providing guidance for which site occupations and Li content levels are accessible to this crystal system.

1.4. Comment #4

RC: ***More details regarding the multiple linear regression (MLR) procedure should be provided.***

AR: We appreciate the recommendation to clarify a major part of this work and have added its own subsection to the supporting information and corresponding link in the text

> The DFT energies were paired with a numerical representation of the structure and fit using a multiple linear regression model (MLR). The structures were represented by a frequency occurrence list of all possible Li-Li interactions. This representation is a reformulation of the connectivity matrix of the Li sub-lattice used for the symmetry reduction described above. The data were split into test and training sets with a 1:3 ratio. For further details about this methodology we refer the reader to SI section X

Where SI section S8 is a new section in the supporting information

> The multiple linear regression scheme works on the basis of finding the best linear fit using the residual sum of squares (RSS)[37] for a given set of input variables x_i, which form a 1-dimensional vector, to predict an output variable y. The relationship that the vast majority of computational research wishes to establish is one between the atomic positions of a system and its energy. Often it is the case that a linear relationship cannot be established, or at least the number of required input parameters to find a general scheme is too large and complex to effectively define. However, when we constrain the total atomic subspace down to a group of very similar systems that are dependent on easily defined variables (such as the occupancy of predefined sites) then a multidimensional linear relationship between a geometric description and energy can be made. For our system, to reduce the required information for each structure to a vector while carrying as much information we perform the routine summarised in figure S7

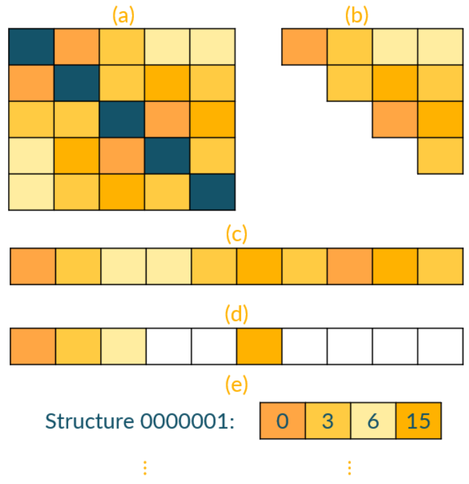

Figure S7: the routine we perform to convert the 3-dimensional coordinates of a string of numbers to a single vector that retains enough information about the system to be predictive. We use a 5×5 Li sublattice as an example here. (a) represents the distance matrix of the periodic Li sublattice, (b) is the upper triangular matrix of a, (c) is the flattened version of b, (d) is all the unique values of c, and (e) is a tally of the number of times this distance occurs in a real, disordered crystal

This routine gives, by necessity, a unique vector for each structure. We find for 120 Li sites there are 86 types of possible Li-Li distance. Meaning each structure can be represented as a vector of 86 length where each number in that vector represents the number of Li-Li interactions occurring Having prepared the data, we can now turn to training our model. We chose to run DFT calculations on two separate types of data sets, one set focuses on the low energy structures as predicted by COMPASSIII (c.f. section S6) which are only 8:48. This ensures there is proper sampling over small deviations in the input vectors. The second set, which is larger, are random samples across all of the configuration space we generate this ensures the model has encountered a suitable breadth of structure types. Finally, we can split the test and train set with a ratio of 1:4. The MLR model then creates a linear fit in 86 dimensions to the training data. We show in the main text that when applied to the test set the model is able to reproduce DFT energies with a Pearson coefficient of 0.9996. We provide examples of how the increasing dimensions of an MLR fit greatly improve the predictive power of the model in figure S8

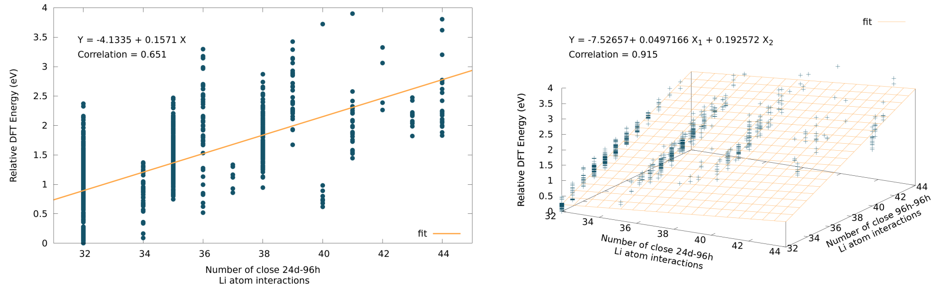

(a) (b)

Figure S8: How increasing the dimension of the input variable with relevant input parameters can greatly improve the predictive power of an MLR model. The yellow line/grid represents the MLR fit in both. (a) is an MLR fit with only one Li-Li interaction tally variable, (b) is an MLR fit with two Li-Li interaction tally variables

We also acknowledge no feature engineering was performed here. This meant we did not remove highly correlated or constant input values, we also didn't normalise our values so that every interaction had an equal weighting. However, given the impressive predictive power of our current model, we found these steps to be unnecessary to achieve the desired results but would recommend implementing them for anyone trying to improve upon our work.

1.5. Comment #5

RC: ***The exact force-field parameters used in the study (Fig. S2), particularly COMPASSIII (which gives the highest agreement with DFT results). Authors cite the original COMPASSIII reference, which is on carbon materials.***

AR: The reviewer is correct in that the COMPASSIII forcefield performs surprisingly well in this context, in fact, it gives the best agreement to DFT among the easily available forcefields. However, this discussion was included mainly as an aside to demonstrate that standard classical force fields do not seem sufficient to describe the structural subtleties of the Li positions, and ultimately we ended up abandoning this line of argument in favour of a machine learned multiple linear regression model which is discussed in the main text. As a consequence, none of the main results in this paper rely on COMPASSIII or on any other force field. We now explain this in a bit more detail in the supporting information. For this reason, we feel that the example is sufficiently well explained and the paper stands on its own without publishing the full parameterization of this side argument.

We make the following edits to the text

Due to the limited number of forcefields available with La and Zr parameters and that we predict the majority of the energy differences to come from the unique Li configurations we believed that excluding non-Li elements may still be sufficient to energetically order our structures. Pure electrostatic summation does not appear to be sufficient in this case (c.f. figure S2(g)), which is why additional non-bonded terms such as van der Waals interactions appear necessary. The most successful method we found was COMPASSIII when applied only to the Li sublattice. ~~The only appropriate method we~~

> ~~found was the COMPASSIII forcefield when applied to only the Li-sublattice (c.f. figure S2h)~~. It was able to order the 13:43 with a correlation coefficient of 0.991. ~~Given the success of COMPASSIII we sought to use it on a wider range of structures (c.f. figure S3).~~

and

> Figure S3 shows that COMPASSIII, when only evaluated for Li atoms, predicts a more pronounced separation of energies depending on the $24d$:$96h$ ratio than is predicted by ONETEP, while good intra-ratio ordering is retained. ~~This issue was considered too large to surmount in this paper and we developed the multiple linear regression model presented in the main text instead.~~ This is the best we could do with easily available force fields, but ultimately insufficient for structural ordering. For this reason, we developed the multiple linear regression model. No results in the main part of the paper are actually built on COMPASSIII.

1.6. Comment #6

RC: ***Not so clear why the calculations on the Li dimer in vacuum have been performed.***

AR: We thank the reviewers for pointing out this lack of clarity. The primary purpose of this calculation was to establish the bond distance at which Li sits according to our DFT set up so we could add that as a line in figure 1d as well as to demonstrate the energetic unfavourability of Li atoms getting within the cutoff distance used for the geometry constraints throughout our manuscript.

We modified our supporting information to make this clearer

> To find the optimal inter atomic distance of a Li-dimer we performed 30 single-point calculations in a large unit cell to prevent self-interaction. We use the same, relevant, settings as used in SI section S7 These calculations were performed to generate the 'Li Dimer' quantity in figure 1d as well as to demonstrate the relative energy penalty for bringing Li atoms as close together as 1.7 Å

1.7. Comment #7

RC: ***For disordered materials, configurational entropy is a major factor that defines the relative stability of a specific atomic configuration/structure. Authors mention that they have excluded these entropic effects altogether without giving much justification or insights into how the final set of lowest-energy structures will be affected by this exclusion.***

AR: We thank the reviewer for bringing this to our attention. This comment has significantly improved the quality of the paper. We had expected the configurational entropy to be small and homogenously spread so unlikely to affect the overall energetic ordering, particularly as we do not consider dopant effects. However, we did find small but relevant inhomogeneities in the distribution of configurational entropy particularly towards the low-energy end of the configuration distribution which had an impact on our results.

We have modified the text to include how the configurational entropy was accounted for.

> ...Further details including all input files can be found in SI sections S1 and S7.
>
> Because some structures have Li distributed in such a way that non-identity symmetry operations are possible there will be variation in the contribution of configurational entropy to the total energy. We

calculated the configurational entropy for all structures and have included it in all results presented in this work, unless stated otherwise. We discuss how we calculated configurational entropy in SI section Y

and created the following SI section

The number of configurations that contribute to configurational entropy for each individual structure can be calculated as the number of symmetry operations (the order) belonging to the Ia3d (96) space group which is then divided by the order of the distributed Li structures.

$$\frac{O_{\text{Ia3d}}}{O_{\text{Li Sublattice}}} = \Omega \quad (1)$$

We can then use this number in the following equation to get the configurational entropy, S_{config}

$$-TS_{\text{config}} = -k_B T \ln(\Omega) \quad (2)$$

Where T is the temperature and k_B is the Boltzmann constant. The vast majority of structures do not have any symmetry operations other than the identity so belong to space group P1. However, some structures have more than the identity operator available to them and will therefore have different entropic contributions. For this reason, the configurational entropy will need to be considered when energetically ordering our structures. We find structures with space group orders of 1, 2, 4, and 8 (c.f. figure S7).

Table 1: All space group orders we recorded and their configurational entropic contributions at 298.15 K

Space group order of the Li sublattice	Number of occurrences	Configurational Entropy Contribution at Room Temperature (eV)
1	2154949	-0.1173
2	3635	-0.09946
4	174	-0.08165
8	14	-0.06384

Table 1 shows that the maximum energy difference that can occur due to configurational entropy is 53 meV. We can also see that only 0.18% of the structures are going to have a different S_{config} than the majority, therefore ordering will be minimally affected.

Because the greatest changes in energy, relative to other structures, occur for high symmetry sites and in figure S7 shows a slight tendency for lower energy structures to have a higher symmetry we do find some

rearrangement occurring towards the low energy portion of our energetic ordering. Specifically, we find that another structure falls within the 1 kT at room temperature range. We have modified figure S9 to reflect this:

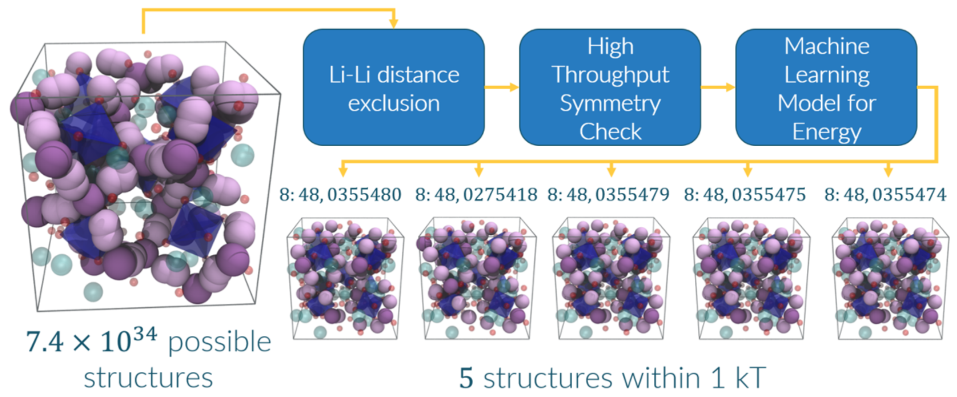

Figure S9: The workflow we employ to find our ~~4~~5 final structures from given occupancies. Above each structure is its respective 24*d*:96*h* ratio and unique identity number in the c-LLZO database we have built (c.f. SI section S1)

changed the relevant conclusions

> All structures are found to occur within a range of ~~5.8~~5.7 eV of our lowest energy structure. ~~19~~17 out of the 20 lowest energy structures have a ratio of 8:48 (c.f. Figure S10b) with the 17th, 19th, and 20th lowest being 9:47. The average energy for all structures occurs at 1.47 eV higher than the ground state structures. The average energy for each ratio increases with increasing 24*d* occupancy, indicating an energetic preference to avoid 24*d* occupation where possible. There are ~~four~~five structures, all 8:48, within 0.026 eV (1 kT at 298 K) of the lowest energy structure we find (c.f. Figure S9). The ~~four~~five lowest energy have very similar atomic coordinates, all have the same 24*d* configuration with only slight variations in the 96*h* configuration, except for the fourth lowest structure which has different 24*d* and 96*h* strucutres. We perform geometry optimisation calculations on the final ~~four~~5 structures and find the energy gap between them narrows further.

and modified the type of assumption we now have to make

> We note that our energetic ordering procedure does not include vibrational entropic contributions and assumes a reasonable retention of ordering upon geometry relaxation, which are both approximations. We have tested the effect of geometry relaxation on a small dataset of 20 structures (c.f. SI section S12). A large-scale energy prediction of the geometry optimised structures falls outside the scope of this communication.

We don't apply the changes to figure S10 because this was mostly to verify the MLR model results and the model does not take into account entropic contributions we modify the caption to make this clear

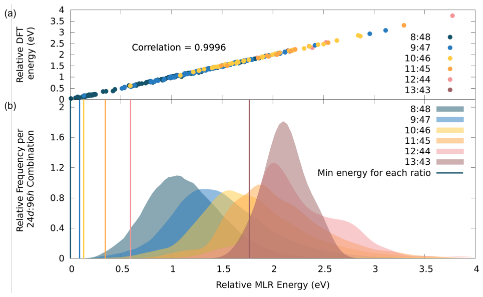

Figure S10: (a) the parity plot of the test set of ONETEP total energies compared to MLR predicted energies for each 24*d*:96*h* ratio. (b) The relative frequency of energy occurrences for each 24*d*:96*h* ratio.Configurational entropic contributions are not included in the energies here

Finally, we modify the text in section S10

Each generated c-LLZO structure will have a symmetry that is a reduced version of the *Ia3d* space group of the general crystal. We have assigned the new space groups for all 2×10^6 symmetry unique structures, knowing the group we can plot the order of the group against the predicted multiple linear regression (MLR) energy. Configurational entropy has not been considered here.

1.8. Comment #8

RC: ***The conclusion given in the abstract "By considering the geometrical constraints that emerge from this methodology we determine that a large portion of previously reported structures may not be feasible or stable." Is not supported by the current results (considering that they do not contain the chemical dopants, as opposed to the literature reports cited here) and hence this statement is probably not valid.***

AR: We thank the reviewer for bringing this to our attention. However, we strongly disagree on this point. A major finding of this work is that experimental and theoretical works regardless of dopant are working with or reporting poor c-LLZO structures. We have accounted for chemical dopants that occur in the 96*h*, 24*d*, and 48*g* sites in figure 1 and tables S1 and S2. In order for the structures reported to exist there must exist a case whereby Li gets closer than 1.7 Å to another Li or a dopant occupying a Li site. This is closer than any reported literature values we can find, for Li-Li or for Li-dopant interactions.

1.9. Comment #9

RC: ***The assumption that the LaZrO structure does not change much with the distribution of the LI atoms within the lattice has limited validity, as the LaO8 and ZrO6 environments are shown to be affected by the immediate Li neighbors (Karasulu et al. 2020 and references therein)***

AR: We agree with the reviewer on this point and have made it clearer in the text that this is a major assumption made in our model for energetic prediction as well as citing the Karasulu et al.. This is unfortunately one of the major drawbacks to working with pure crystallographically predicted structures. We do already acknowledge this in our conclusions and provide steps to overcoming this in future work.

Because we are working with only the crystallographically predicted structures ~~we assume~~ the base LaZrO structure remains ~~relatively~~ unchanged with each configuration and therefore ~~the majority of~~ all energetic changes are due to Li placement. We acknowledge that in reality, the geometry does change considerably depending on the Li environment[22]. However, this is a suitable assumption to make to energetically order the crystallographically predicted structures we generate. Therefore, an expression of all types of Li interactions would be a sufficient descriptor for predicting the energies of c-LLZO structures. The DFT energies were paired with a numerical representation of the structure and fit using a multiple linear regression model (MLR). The structures were represented by a frequency occurrence list of all possible Li-Li interactions. This representation is a reformulation of the connectivity matrix of the Li sub-lattice used for the symmetry reduction described above. The data were split into test and training sets with a 1:3 ratio.

1.10. Comment #10

RC: ***References need to be edited as many typos and formatting errors are visible, particularly ref 30.***

AR: We thank the reviewer for the thoroughness and have now checked all references and ensured they are properly formatted.

1.11. Comment #11

RC: ***p. 21: 'All energies are relative to the 355474 result for each calculation type' sentence is not clear.***

AR: We appreciate the reviewer for catching this and have now improved the clarity

All energies are relative to the ~~355474 result for each calculation type~~ lowest MLR energy structure.

1.12. Comment #12

RC: ***Fig S6: not so clear which data points correspond to the SP and GO data sets.***

AR: We thank the reviewer for their observation and have improved the caption. Single point energy and geometry optimisations were performed for all structures and do not belowng to different data sets.

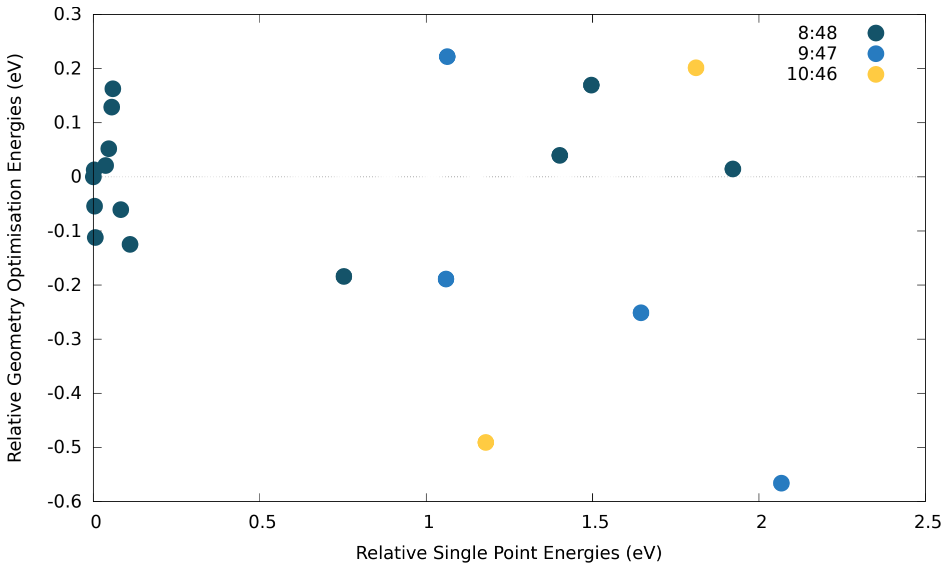

Figure S11: A comparison of the energetic ordering of 20 structures (10 lowest structures according to MLR and 10 random structures). Single point and geometry optimisation calculations were performed using DFT for all structures. All energies are relative to the ~~355474 result for each calculation type~~ lowest MLR energy structure.

1.13. Comment #13

RC: ***Fig. S5: How is the symmetry group order is defined?***

AR: We thank the reviewer for highlighting this lack of clarity. We define the order of a space group as the number of unique symmetry operations a space group has. We have added this definition to the relevant supporting information section.

> Each generated c-LLZO structure will have a symmetry that is a reduced version of the *Ia3d* space group of the general crystal. We have assigned the new space groups for all 2×10^6 symmetry unique structures, knowing the group, we can plot the order of the group against the predicted multiple linear regression (MLR) energy. The order is the number of unique symmetry operations belonging to an individual space group.

2. Notes From the Journal

2.1. Comment #1

RC: ***Please include annotated version(s) of your revised publication file(s) with colored text or highlights indicating the revisions that you have made, and upload them as "Supporting Information for Review Only." Please also upload "clean" copies for publication. (No highlighting, annotations, or colored text permitted.)***

AR: We have attached both copies with the naming convention provided. The clean version requested is labelled 'main_manuscript'

2.2. Comment #2

RC: ***Abstract: Shorten the abstract to 150 words or fewer.***

AR: We have done so

~~Experimental and theoretical works have, to date, been unable to uncover the ground state configuration of prominent solid electrolyte candidate cubic $Li_7La_3Zr_2O_{12}$ (c-LLZO). Computational studies rely on an initial low-energy structure as a reference point. In this study, we present a methodology to identify energetically favourable configurations of c-LLZO, enabling the isolation of low-energy structures, for a crystallographically predicted structure. We begin by eliminating structures that involve overlapping Li atoms based on nearest neighbour counts. We further reduce the configuration space by eliminating symmetry images from all remaining structures. This is followed up with a machine learning-based energetic ordering of all remaining structures. By considering the geometrical constraints that emerge from this methodology we determine that a large portion of previously reported structures may not be feasible or stable. The method developed here could be extended to other ion conductors and partially occupied crystal sites. Furthermore, we provide all structures generated in a freely accessible database with the aim to improve accuracy and reproducibility in future c-LLZO research.~~Experimental and theoretical works have, to date, been unable to uncover the ground state configuration of solid electrolyte candidate cubic Li7La3Zr2O12 (c-LLZO). Computational studies rely on an initial low-energy structure as a reference point. We present a methodology to identify energetically favourable configurations of c-LLZO for a crystallographically predicted structure. We begin by eliminating structures that involve overlapping Li atoms based on nearest neighbour counts. We further reduce the configuration space by eliminating symmetry images from all remaining structures. We then use a machine learning-based energetic ordering of all remaining structures. By considering the geometrical constraints that emerge from this methodology we determine that a large portion of previously reported structures may not be feasible or stable. The method developed here could be extended to other ion conductors. We provide a database of all structures generated with the aim of improving accuracy and reproducibility in future c-LLZO research.

2.3. Comment #3

RC: ***TOC Graphic: Provide a TOC image per journal guidelines (2 in x 2 in; on the same page as the abstract) with the heading "TOC Graphic" above the graphic. The graphic should be in the form of a structure, graph, drawing, photograph, or scheme—or a combination. Non-scientific cartoon-like images or caricatures are discouraged.***

AR: We have made a TOC and placed it on the same page as the abstract

TOC Graphic

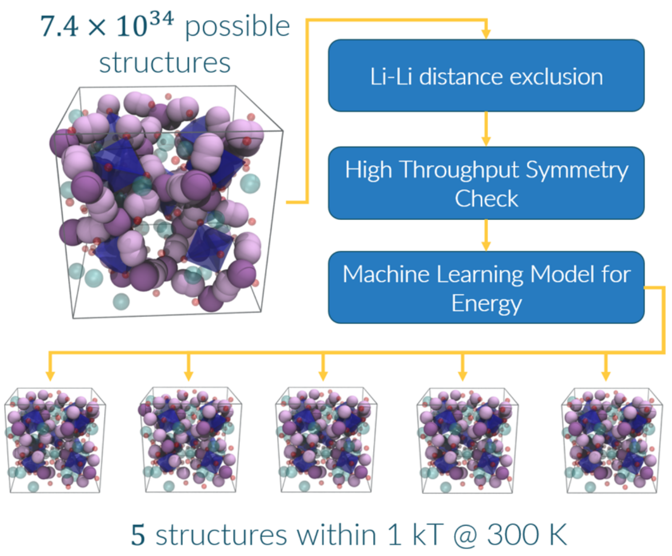

2.4. Comment #4

RC: ***Headers: Please remove "Maint Text" header.***

AR: Done

2.5. Comment #5

RC: ***Figures: Please label the parts of Figure 2 as they are mentioned in text.***

AR: The figure has now been updated

TOC Graphic

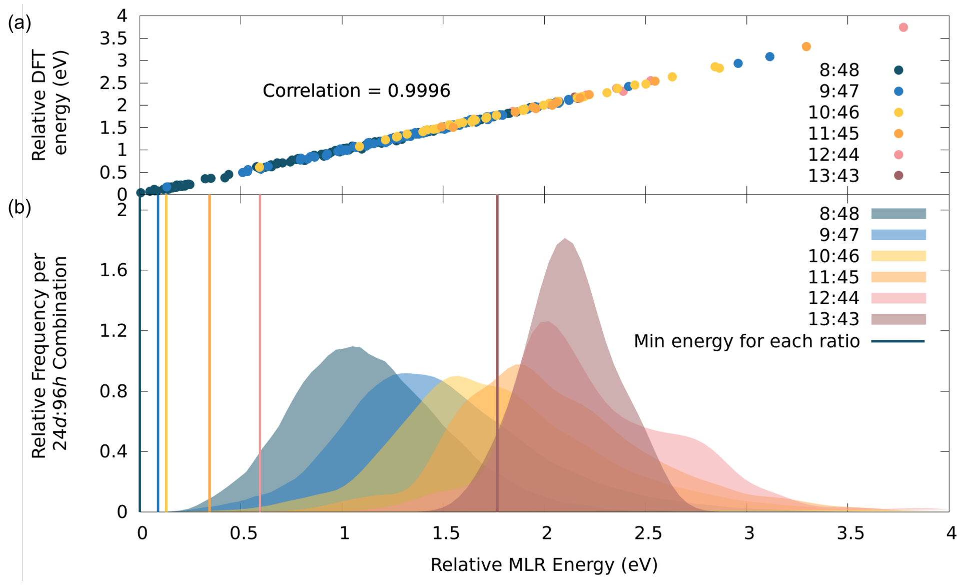

2.6. Comment #6

RC: ***References: In both the main file and the supporting information, fix the style of all references to use JPCL formatting (check all references carefully). ***JPC Letters reference formatting requires that journal references should contain: () around numbers; author names; article title (titles entirely in title case or entirely in lower case); abbreviated journal title (italicized); year (bolded); volume (italicized); and pages (first-last). Book references should contain author names; book title (in the same pattern); publisher; city; and year. Websites must include date of access.***

AR: We have checked all references now and found no errors in them and are using the referencing style according to this journal's latex template
